# Nurse-initiated transitional care interventions to support the transition from hospital to home amongst older stroke survivors and caregivers: A systematic review and meta-analysis

**DOI:** 10.1177/02692155261430092

**Published:** 2026-06-10

**Authors:** Usanee Ankanawin, Lisa Kidd, Dawn A Skelton

**Affiliations:** 1 School of Health and Life Sciences, 150824 Glasgow Caledonian University, Glasgow, UK; 2 School of Health and Life Sciences, 3525 Research Centre for Health (ReaCH), Glasgow Caledonian University, Glasgow, UK

**Keywords:** Older, stroke survivors, transitional care, hospital-to-home, nurse

## Abstract

**Objective:**

To explore and describe the components and underpinning theories of nurse-initiated transitional (hospital-to-home) care and to evaluate the effects of interventions on activities of daily living (ADLs), quality of life (QoL), depression, anxiety, self-efficacy, stroke-related knowledge, patient satisfaction and healthcare service utilisation in older stroke survivors and self-efficacy, caregiver burden, QoL and satisfaction with care in caregivers.

**Data sources:**

MEDLINE, CINAHL, Cochrane Library, Health and Medical Collection, Nursing and Allied Health, Web of Science, Health Source: Nursing/Academic Edition and Scopus databases were searched in February 2024 and updated in March 2026.

**Review methods:**

Randomised controlled trials (RCTs) and cluster-RCTs were included. Risk of bias was assessed using the Cochrane Risk of Bias tool, and evidence quality was rated with GRADE. Meta-analysis was undertaken using random-effects models.

**Results:**

Seventeen trials were included. Transitional care interventions were guided by various theoretical frameworks and had multiple components. Interventions improved ADLs at 1 month (standardised mean difference (SMD) 0.54; 95% CI 0.12–0.97, four studies) and 3 months (SMD 0.43; 95% CI 0.12–0.74, seven studies), increased patient satisfaction and reduced hospital readmissions up to 3 months. Interventions may improve the Mental Component Score at 1 month, Role Limitations due to Physical Problems and General Health of QoL at 6 months. The certainty of evidence was low to very low.

**Conclusion:**

Nurse-initiated transitional care can improve ADLs and QoL, while reducing hospital readmissions among older stroke survivors during the hospital-to-home transition. Multicomponent interventions combining home visits and follow-up phone calls may enhance improvements in ADLs. High-quality studies are needed to clarify long-term effects.

**PROSPERO ID:**

CRD42024517619.

## Introduction

Stroke is the second leading cause of death, and the third most common cause of disability-adjusted life-years lost among noncommunicable diseases worldwide.^1,2^ Over 93 million people are currently living with stroke.^2^ The global increase in the population aged 60 years and older^3^ is expected to result in a growing number of older stroke survivors, thereby placing a greater burden on health care systems worldwide, particularly in low- and middle-income countries.^1,2^

Older stroke survivors require support from family caregivers to assist with daily activities, functional rehabilitation, medication management, complication prevention, and facilitating social interaction.^4–6^ Among stroke survivors, older adults face a heightened risk of worsened physical function,^7,8^ emotional distress,^5^ adverse events, complications,^9,10^ and hospital readmissions after discharge from the hospital to home.^10–12^ In addition, caregivers also face emotional distress, such as anxiety, fear, and being overwhelmed by the increased responsibility of caring for older stroke survivors at home.^5,13,14^ These issues have become more pronounced due to shortened hospital stays, rushed discharges, limited involvement in discharge planning, and a lack of post-discharge care support.^5,13,14^

Transitional care is defined as ‘a set of actions designed to ensure the coordination and continuity of healthcare as patients transfer between different locations or different levels of care within the same location’.^15^^(p.556)^ Transitional care interventions have been widely recognised as essential aspect of care during the transition from hospital to home following stroke, as the interventions have positive effects on activities of daily living,^16–19^ quality of life^16,17^ and depression.^16,17^ Global stroke guidelines recommend that stroke survivors and their families or carers be actively involved in planning their transfer of care before hospital discharge, receive early supported discharge services and be referred to community stroke rehabilitation when needed, delivered through interprofessional care and collaboration across the healthcare system.^20–22^

Fragmented post-discharge services and a lack of continuing care support after discharge lead to stroke survivors and their caregivers facing challenges such as feeling abandoned in adjusting to their new roles and life journeys.^13^ The literature highlights that effective transitional care programs are interventions designed to bridge the care gap between hospital and home transitions, initiated during the hospital stay and continuing into the post-discharge period.^23^ Whilst there have been multiple systematic reviews considering transitional care and stroke survivors,^16–19^ there are none which explicitly consider transitional care initiated during pre- and continuing post-discharge in older stroke survivors, nor the specific role of nurses as the primary providers or coordinators of transitional care interventions. Previous reviews have focused on multidisciplinary rehabilitation professionals^17–19^; however, nurses are widely recognised as having a key role in transitional care for stroke survivors and their family caregivers as they move from hospital to home, particularly in preparing for and supporting self-management, rehabilitation and recovery.^24–28^

This review aims to explore and describe the components and underpinning theories of existing nurse-initiated transitional care interventions and to evaluate the effectiveness of the interventions on activities of daily living, quality of life, depression, anxiety, self-efficacy, stroke-related knowledge, satisfaction with care and the utilisation of health care services as older stroke survivors’ outcomes and caregivers’ self-efficacy, burden, quality of life and satisfaction with care.

## Methods

The protocol of this review was registered in the International Prospective Register of Systematic Review (PROSPERO) (CRD42024517619), and minor amendments to the protocol can be found in Supplemental Table 1. This systematic review and meta-analysis was reported in accordance with the Preferred Reporting Items for Systematic Reviews and Meta-Analyses (PRISMA) 2020 statement guidelines.^29^

The search was conducted in February 2024 and last updated in March 2026 in the following databases: MEDLINE, CINAHL, Cochrane Central Register of Controlled Trials, ProQuest Health and Medical Collection, ProQuest Nursing and Allied Health, Web of Science, Health Source: Nursing/Academic Edition and Scopus. Keyword and subject heading (where relevant) were undertaken for the following three terms: stroke, transitional care and nurse, including considering additional keywords, abbreviations and MeSH terms. A Boolean search was conducted by combining keywords, as shown in Supplemental Table 2 (e.g. stroke* OR “acute stroke*” OR “cerebrovascular accident*” OR “cerebrovascular stroke*” OR “cerebral stroke*” OR “stroke survivor*” OR CVA OR “cerebral infarction”) AND (“transition* care*” OR “transition* home” OR “transition* of care” OR “care* transition” OR “transition*” OR “hospital to home*” OR “discharge plan*” OR “patient discharge*” OR “hospital discharge*” OR discharge* OR “coach* program*” OR “health coach*” OR “caregiver-oriented” OR “holistic care”) AND (nurse* OR nursing OR “nurse-led” OR multidisciplinary OR integrated OR “nurse-coordinated” OR interprofessional*). References of included studies, grey literature and Google Scholar were manually screened for additional studies, and corresponding authors were contacted if additional clarification was required. Studies published in English from 2009 onward were included, as they represent the current body of evidence on transitional care.

The PICOS framework was used to identify eligible studies.^30^ The inclusion criteria were as follows:

*Population:* Stroke survivors aged ≥60 years,^3^ or with a mean age ≥60 in studies including participants of any age; diagnosed with haemorrhagic, ischaemic or recurrent stroke; and discharged from hospital to home, with or without family caregivers.

*Intervention:* Interventions were implemented during both pre-discharge and post-discharge periods to support older stroke survivors and/or caregivers in the transition from hospital to home, lasting up to six months after discharge; initiated by nurses or a multidisciplinary team, with nurses performing at least one of the following tasks: assessing patients’ and/or caregivers’ needs, providing discharge planning, health education, self-management support, health coaching or conducting follow-up after discharge.^24–28^

*Comparison:* Other interventions or usual care.

*Outcome:* Older stroke survivors: activities of daily living, depression, anxiety, quality of life, self-efficacy, stroke-related knowledge and satisfaction with care. Caregivers: self-efficacy, caregiver burden, quality of life and satisfaction with care. Healthcare service utilisation: emergency department visits and hospital readmissions. Measurement: outcomes were assessed at baseline and at all follow-up points after discharge.

*Study design:* Any published randomised controlled trials (RCTs) or cluster-RCTs.

Studies were excluded if the mean age of stroke survivors was under 60 years or if participants were discharged to care homes or nursing care facilities. Interventions delivered only pre- or only post-discharge, those lasting beyond six months post-discharge and those led by multidisciplinary teams without clear delineation of nurses’ roles or involvement were excluded.

All records were imported into Covidence, with duplicates removed by one reviewer (UA). Two reviewers (UA, LK) independently screened titles and abstracts, resolving discrepancies through discussion. Full texts of eligible studies were independently reviewed (UA, LK), with disagreements resolved via discussion or consultation with a third reviewer (DAS). Further clarification was sought from six studies regarding sample details, nursing roles and intervention data. The selection process was documented using a PRISMA flow diagram.^29^

A bespoke data extraction form was used to capture key details from each eligible study (i.e. authors, publication year, country, design, participant characteristics, intervention/comparator details and outcomes). Intervention descriptions followed the Template for Intervention Description and Replication (TIDieR) checklist.^31^ Outcome data were collected at baseline, post-intervention and follow-up (if any). The data extraction form was pilot tested (UA, LK) using two studies, then refined. A subset of studies (n = 4, 24%) underwent independent double extraction by two authors (UA and LK or DAS), with discrepancies resolved through discussion. The remaining 13 studies were extracted by one author (UA).

Risk of bias was assessed using the Cochrane Risk of Bias 2 tool for randomised trials (RoB2)^32^ and its extension for cluster-RCTs (RoB 2 CRT).^33^ Two authors (UA and LK or DAS) independently assessed four studies, resolving discrepancies through discussion or third-author arbitration if needed. One author (UA) conducted initial quality assessments for the remaining studies, with another (LK) verifying judgements and any disagreements resolved by the third author when necessary. Certainty of evidence for each outcome was rated using GRADE (high, moderate, low, or very low)^34^ by one author (UA) and checked by a second (LK).

A narrative synthesis was used to summarise study characteristics, participant demographics, intervention components and underpinning theories. Meta-analysis was undertaken using Review Manager 5 (RevMan5) when data from two or more studies were available.^35^ For duplicate reports of the same study, only one dataset was used. Treatment effects were assessed post-intervention and at follow-ups^36^ (1, 3 and 6 months). Where standard deviations were missing, they were calculated from standard errors^36^ using RevMan5. Mean differences (MDs) with 95% confidence intervals (CIs) were pooled for continuous outcomes, while standardised mean differences (SMDs) were used when multiple measures assessed the same outcome.^36^ Risk ratios (RRs) with 95% CIs were calculated for dichotomous outcomes (e.g. hospital readmission). Due to the complexity and heterogeneity of the intervention characteristics, it was anticipated that the effects of the interventions would vary across studies, making it unlikely that a single common effect size could be assumed. A random-effects model was chosen to estimate the expected distribution of intervention effects. CIs were estimated using the Wald-type method.^35^ Heterogeneity was assessed using Tau^2^, I^2^ and forest plots.^35^ Restricted Maximum Likelihood was used to estimate variance.^35^ Sensitivity analysis excluded studies with high or unclear risk of bias to evaluate the robustness of the findings in a meta-analysis. Post-hoc subgroup analyses explored the impact of intervention duration, theory, contact mode, provider type, contact intensity and number of components on activities of daily living and readmission rates. Selective outcome reporting was evaluated by comparing the analyses pre-specified in the study protocol or outcome measures and analyses listed in the methods section of each study with those published.^37^ A funnel plot was employed to visually assess publication bias when the meta-analysis included ten or more studies.^37^

## Results

The search identified 11,888 records. After duplicates were removed, 7441 records were screened for titles and abstracts. Of these, 7338 records were removed because they did not meet the review criteria, and two full-text articles could not be retrieved. The remaining 101 reports, along with an additional three reports identified through reference lists of included studies, underwent full-text screening for eligibility. Eighty-four reports did not meet the inclusion criteria. In total, there were 20 articles reporting on 17 trials included in this systematic review (Figure 1).

**Figure 1.**
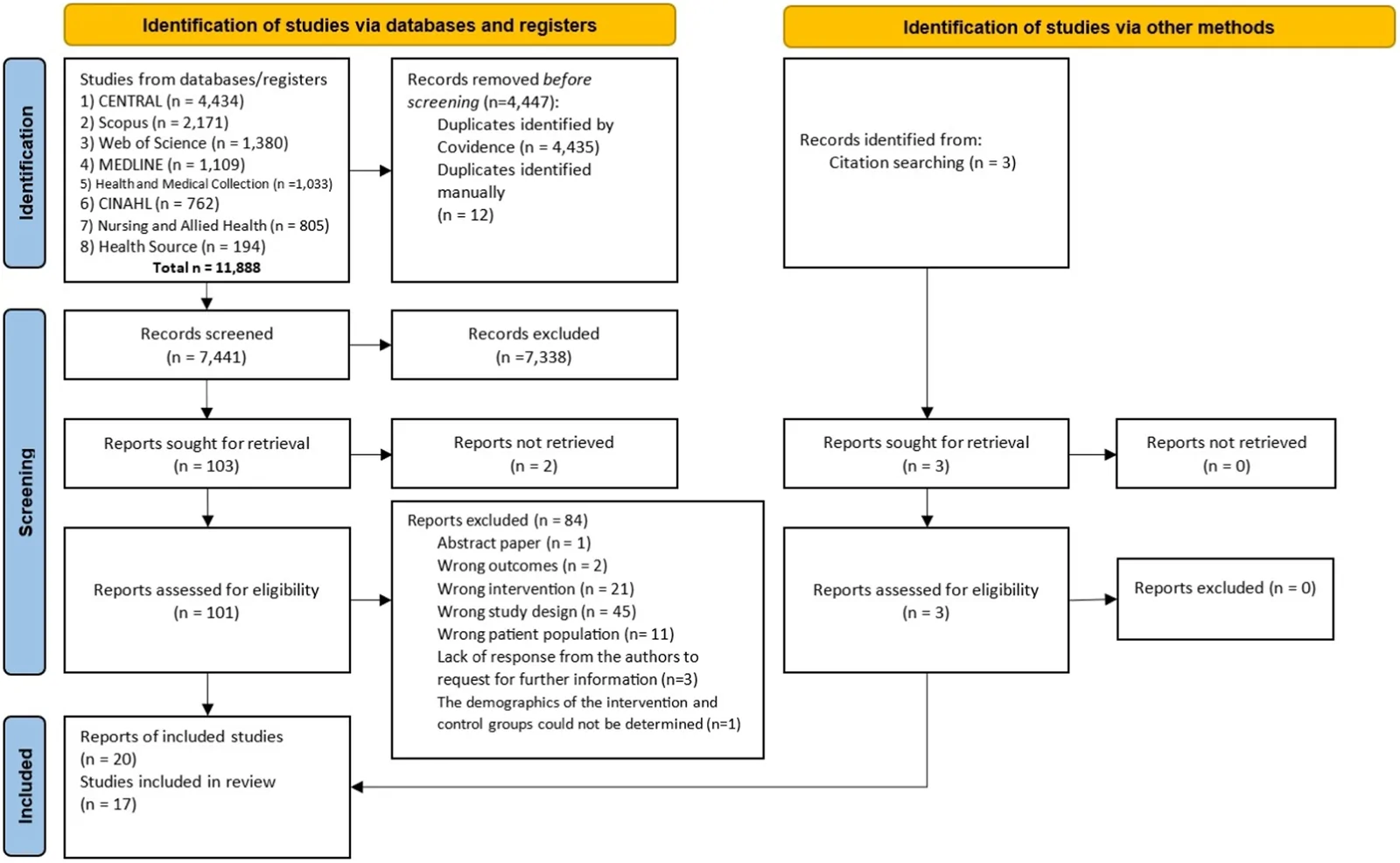
PRISMA flow diagram of search process.

The risk of bias assessments for the included studies are summarised in Figure 2 and Supplemental Tables 4 and 5. Six studies were assessed as having a low risk of bias,^25,28,38–41^ while some concerns were noted in six studies,^26,42–46^ and five studies were assessed as having a high risk of bias.^27,47–50^ The main methodological issues identified were as follows: several trials reported unclear details regarding randomisation methods, inconsistencies in measurement time points, and insufficient information about missing outcome data. Four included studies reported outcomes according to a prespecified plan for outcome measures and analyses, as outlined in pre-trial protocols,^27,39–41^ and outcomes reported in 13 studies followed the plan listed in the methods section. None of the selected studies employed a double-blind design, owing to the nature of the interventions, which focused on health-related behaviour change and relied on patient-reported outcome measures. Funnel plots could not be used to evaluate publication bias due to the small number of included studies in the meta-analysis.^37^

**Figure 2.**
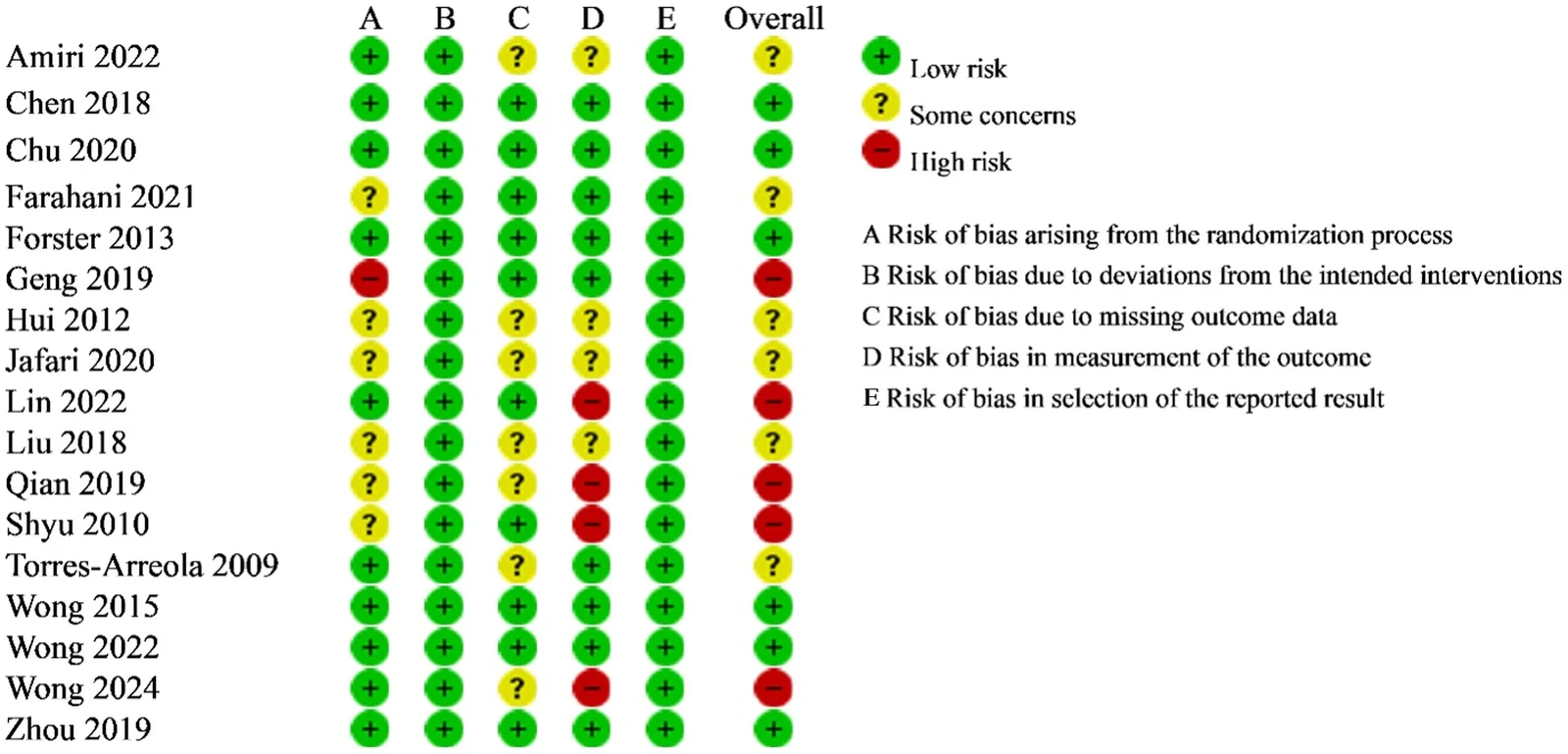
Risk of bias of included studies.

The details of the study characteristics are shown in Table 1 and Supplemental Table 3. Sixteen studies were RCTs, and one study was a cluster-RCT.^40^ Eleven studies were conducted in China,^25,27,28,38,39,41,43,45,47,48,50^ three in Iran,^26,42,44^ one in Taiwan,^49^ one in UK^40^ and one in Mexico.^46^ Twelve studies recruited solely stroke survivors, while five studies recruited both stroke survivors and caregivers.^26,27,40,41,49^ Participants were recruited from stroke units or neurological wards (n = 13) and acute stroke care settings (n = 4). The sample sizes of stroke survivors ranged from 20 to 478 in each group, with a total of 1276 intervention stroke participants and 1264 stroke participants in the control groups. The mean age of stroke survivors ranged from 60.20 (4.35) to 76.32 (4.56) years. Nine studies had participants with both ischaemic and haemorrhagic stroke, and two studies had only ischaemic patients, while six studies did not report the type of stroke.^26,28,45,46,48,50^ In 11 studies, interventions were delivered solely by nurses, whereas in six studies,^28,40,42,45,47,50^ they were provided by nurses in collaboration with other multidisciplinary team members (i.e. medical staff, psychologists, physiotherapists and social workers). Interventions were initiated 48–72 h after admission (n = 3),^44,46,49^ after patients’ conditions had stabilised (n = 9),^26–28^^,38–41,48,50^ prior to discharge (n = 5),^25,42,43,45,47^ and continued into the post-discharge period (n = 17), lasting 2 weeks to 6 months with follow-up periods of 2 weeks to 12 months. The most frequently reported outcomes were activities of daily living (n = 11) and quality of life (n = 11). Intervention materials of the included studies were brochures for exercise programs, educational booklets, videos, training manuals, a stroke self-management home care toolkit, and a stroke health coaching diary. Control groups received standard or usual care, which was articulated differently across studies, including discharge planning, health education, rehabilitation training, follow-up and home visits.

**Table 1.** Characteristics of included studies.

First author, year, country; design	Number of participants per group; age (mean(SD))	Intervention name	Intervention length (weeks or months) and frequency	Outcome measure (scale)
Amiri, 2022,^42^ Iran; RCT	Stroke survivors IG 36; 69.1(8.64) CG 36; 67.67(8.30)	5A self-management model	7 weeks; 1 individualised assessment and advice, 4 virtual training, 1 telephone follow-ups	Stroke self-efficacy (SSEQ)
Chen, 2018,^38^ China; RCT	Stroke survivors IG 72; 65.92(12.80) CG 72; 64.78(9.87)	Patient-Centered Self-Management Empowerment Intervention	6 weeks; 5 individual and 1 small group teaching sessions (2 weeks), weekly telephone follow-ups (4 weeks)	ADLs (BI), QoL (SF-36), Depression (CES-D), Stroke self-efficacy (SSEQ), Re-hospitalisation
Chu, 2020,^39^ China; RCT	Stroke survivors IG 31; 62.84(9.71) CG 30; 66.17(8.51)	Nurse-Trained, Family Member-Delivered Rehabilitation Model	8 weeks; 3 rehabilitation training sessions, telephone follow-ups week 2, 4, 8	ADLs (BI), QoL (EQ-5D), Caregiver burden (CBI)
Farahani, 2021,^26^ Iran; RCT	Stroke survivors IG 58; 68.5(13.16) CG 58; 64.65(12.2) Caregivers IG 58; 43.98(13.8) CG 58; 43.41(11.25)	Supportive home care programme	2 weeks; 2 educational sessions, and home visits	Caregiver burden (CBI)
Forster, 2013,^40^ UK; Cluster-RCT	Stroke survivors IG 450; 71.0(12.76) CG 478; 71.3(12.18) Caregivers IG 450; 61.1(14.64) CG 478; 60.8(13.91)	London Stroke Carers Training Course Structured training	6 months; 14 Structured training items (6 items mandatory and 8 items based on individual needs), one follow-up visit/phone call	Functional independence (NEADL, BI), QoL (EQ-5D), Stroke Impact Scale (SIS), Anxiety (HADS-A), Depression (HAD-S), Caregiver burden (Caregivers burden scale), Caregiver QoL (EQ-5D)
Geng, 2019,^47^ China; RCT	Stroke survivors IG 30; 67.6(4.3) CG 30; 68.9(4.4)	Integrated Behavioral Model based Transitional care	12 weeks; 1 bedside health education, weekly home visits and telephone follow-ups	ADLs (MBI), QoL (SF-36), Stroke knowledge (Stroke knowledge questionnaire)
Hui, 2012,^43^ China; RCT	Stroke survivors IG 27, CG 26; 63.41(11.08) (overall mean age)	Home visits nursing	3 months; 1 discharge instruction, weekly home visits in the first month, biweekly home visits in the second month, once in the third month	ADLs (MBI)
Jafari, 2020,^44^ Iran; RCT	Stroke survivors IG 30; 60.70 (7.33) CG 30; 60.20 (4.35)	Home-Based Rehabilitation	2 weeks; 3 education sessions, telephone follow-ups twice a week	QoL (SS-QOL-49)
Lin, 2022,^27^ China; RCT	Stroke survivors IG 70; 60.8(11.0) CG 70; 62.5 (10.9) Caregivers IG 70; 54.5(11.9) CG 70; 53.8(11.0)	Nurse-led health coaching programme	12 weeks; 5 coaching sessions, weekly telephone support, bi-weekly face-to-face coaching	QoL (SSQoL-12), Self-efficacy (SSEQ), Stroke-related health knowledge (SPKQ), Unplanned hospital readmissions, Caregiver-related burden (Modified Caregiver Strain Index)
Liu, 2018,^45^ China; RCT	Stroke survivors IG 20; 76.32(4.56) CG 20; 74.12(3.12)	Nurse-led transitional care intervention	4 weeks; 1 discharge instruction, home visit and telephone follow-ups every day	QoL (SF-36)
Qian, 2019,^48^ China; RCT	Stroke survivors IG 35; 66.54(10.16) CG 37; 64.8(10.49)	Transitional nursing care mode	5 weeks; 1 small class health education, telephone follow-ups (week 1), home visits (week 4)	ADLs (BI), Patient satisfaction with nursing care (PSHC), Frequency of hospitalisation health service, Frequency of emergency health service
Shyu, 2010,^49^ Taiwan; RCT	Stroke survivors IG 72; 74.1(6.5) CG 86; 74.2(6.5) Caregivers IG 72, CG 86; 47.9 (13.7) (overall mean age)	Family caregiver-oriented intervention programme	4 weeks; 4–5 discharge instructions, 1 telephone call in the first week, home visits in week 1, 4	ADLs (Chinese BI), QoL (SF-36), Hospital readmission rate, Caregiver health-related QoL (SF-36)
Torres-Arreola, 2009,^46^ Mexico; RCT	IG 59; 69.4(12.0) CG 51; 69.8(8.8)	Nurse-directed rehabilitation programme	4 months; daily education and physical therapy, daily home visits (2 weeks), bi-weekly home visits (2 weeks), weekly home visits (3 months)	ADLs (BI)
Wong, 2015,^25^ China; RCT	Stroke survivors IG 54; 67.5(11.6) CG 54; 71.5(11.6)	Transitional care programme	4 weeks; 1 family meeting, 3 home visits, 1 telephone follow-up a week	ADLs (MBI), QoL (SF-36, WHO-QOL-SRPB), Depression (CES-D), Patient satisfaction (PSQ-HK), Hospital readmission, Emergency department attendance rate
Wong, 2022,^28^ China; RCT	IG 58; 66.21(10.07) CG 58; 67.00(8.61)	Structured 4C, home-based post-discharge program	12 weeks; 1 family meeting, home visits and telephone follow-ups 5 days a week	ADLs (MBI), QoL (EQ-5D-5L), Stroke Impact scale (SIS), Self-Efficacy (SEMCD-6)
Wong, 2024,^50^ China; RCT	Stroke survivors IG 58; 66.21(10.07) CG 58; 67.00(8.61)	Enhanced post discharge home-based care program intervention	12 weeks; 1 inpatient visit, 1 coaching session, 4 home visits, 4 reinforcement calls, 4 telephone follow-ups, 24-h hotline	Readmission episode, Unplanned emergency service
Zhou, 2019,^41^ China; RCT	Stroke survivors IG 116; 64.3(9.2) CG 128; 66.2(9.4) Caregivers IG 116; 51.4(12.3) CG 128; 52.3(12.8)	Rehabilitation Through Caregiver- Delivered Nurse-Organized Service Programs	8 weeks; 2–3 rehabilitation training sessions, telephone calls week 2, 4, 8	ADLs (BI), QoL (EQ-5D), Caregiver burden (Caregiver Burden Index)

RCT: randomised control trial; IG: intervention group; CG: control group; SD: standard deviation; ADLs: activities of daily living; BI: Barthel Index; CBI: Caregiver Burden Inventory; CES-D: Center of Epidemiological Studies for Depression Scale; EQ-5D: EuroQol five dimensions questionnaire; EQ-5D-5L: 5-level EQ-5D version; HAD-S: Hospital Anxiety and Depression Scale; HADS-A: Hospital Anxiety and Depression Scale – Anxiety; MBI: Modified Barthel Index; NEADL: Nottingham Extended Activities of Daily Living Scale; PSHC: Patient satisfaction with hospital care; QoL: Quality of Life; SEMCD-6: Self-Efficacy for Managing Chronic Disease 6-item Scale; SF-36: Short Form-36; SPKQ: Stroke Prevention Knowledge Questionnaire; SSEQ: Stroke Self-Efficacy Questionnaire; SS-QOL-12: Stroke-Specific Quality of Life Scale 12 items; SS-QOL-49: Stroke-specific quality of life (SS-QOL) scale 49 items; WHO-QOL-SRPB: World Health Organisation – Quality of Life – Spirituality, Religiousness and Personal Beliefs.

Transitional care interventions comprised multiple components, predominantly health education (n = 15, 88%), goal setting and personalised care planning (n = 10, 59%), functional rehabilitation (n = 9, 53%), patient- and caregiver-centred assessment (n = 9, 53%) and self-management (n = 5, 29%) (Supplemental Table 3 and Table 6). Health education was implemented either as a comprehensive package or curriculum,^26,27,40,44,48^ or integrated into transitional care.^28,38,41–43^^,45–47,49–50^ Goal setting and planning were conducted to promote behavioural change, enhance self-care skills^25,27,28,38,42,43,45^ and support physical function recovery.^39,41,50^ Rehabilitation plans and practical rehabilitation training sessions were developed with support from physiotherapists or medical specialists.^27,28,38,39,41,43,45,46,48^ Patient- and caregiver-centred assessment mainly focused on patients’ health status, functional abilities, stroke-related knowledge and health behaviours.^25,28,38,40,42,45,47,49,50^ Self-management was primarily structured around comprehensive case management.^25,28,38,42,50^ Eleven studies reported using underpinning theories or frameworks (Supplemental Table 6), which included the Omaha System,^25,28,48,50^ Transitional Care Framework,^28^ Brunnstrom's Stages of Recovery,^46,48^ Naylor's Transitional Care Model,^25,45^ the Behavioural Model,^47^ the 5A Self-Management Model,^42^ the Health Empowerment Model,^38^ Self-Efficacy Theory^27^ and the Motor Relearning Theory.^41^

The effects of transitional care interventions were assessed based on outcomes for older stroke survivors, caregivers and the utilisation of health care services. The meta-analyses are presented first, followed by a narrative review of findings from studies not included in the meta-analyses.

*Activities of daily living:* Eleven studies assessed activities of daily living. Pooled results showed an effect in favour of the intervention group compared with the control group at 1 month^25,38,43,49^ (RE, SMD 0.54, 95% CI 0.12 to 0.97, I^2^ = 79%, 4 trials, 463 participants) and at 3 months^28,38,39,41,43,48,49^ (RE, SMD 0.43, 95% CI 0.12 to 0.74, I^2^ = 78%, 7 trials, 848 participants) (Figure 3). The certainty of the evidence was rated as very low due to risk of bias, inconsistency and imprecision (Supplemental Table 7). The effect remained significant in the sensitivity analysis after excluding studies with some concerns regarding the risk of bias (Supplemental Figures S2 and S4). There was no effect in favour of the intervention on activities of daily living at 6 months^28,39,41,47,49^ (RE, SMD 0.25, 95% CI −0.05 to 0.55; I^2^ = 70%) (Supplemental Figure 1). The findings indicated that the intervention produced a medium effect on activities of daily living at 1 month and 3 months, but not at 6 months, as determined by the SMD effect size interpreted according to Cohen's rules of thumb (0.2 = small, 0.5 = medium, 0.8 = large).^51^ Two studies not included in the meta-analysis showed no significant improvements in activities of daily living at 6 months^40,46^ and 12 months (MD 0.2, 95% CI −0.5 to 0.8).^40^

**Figure 3.**
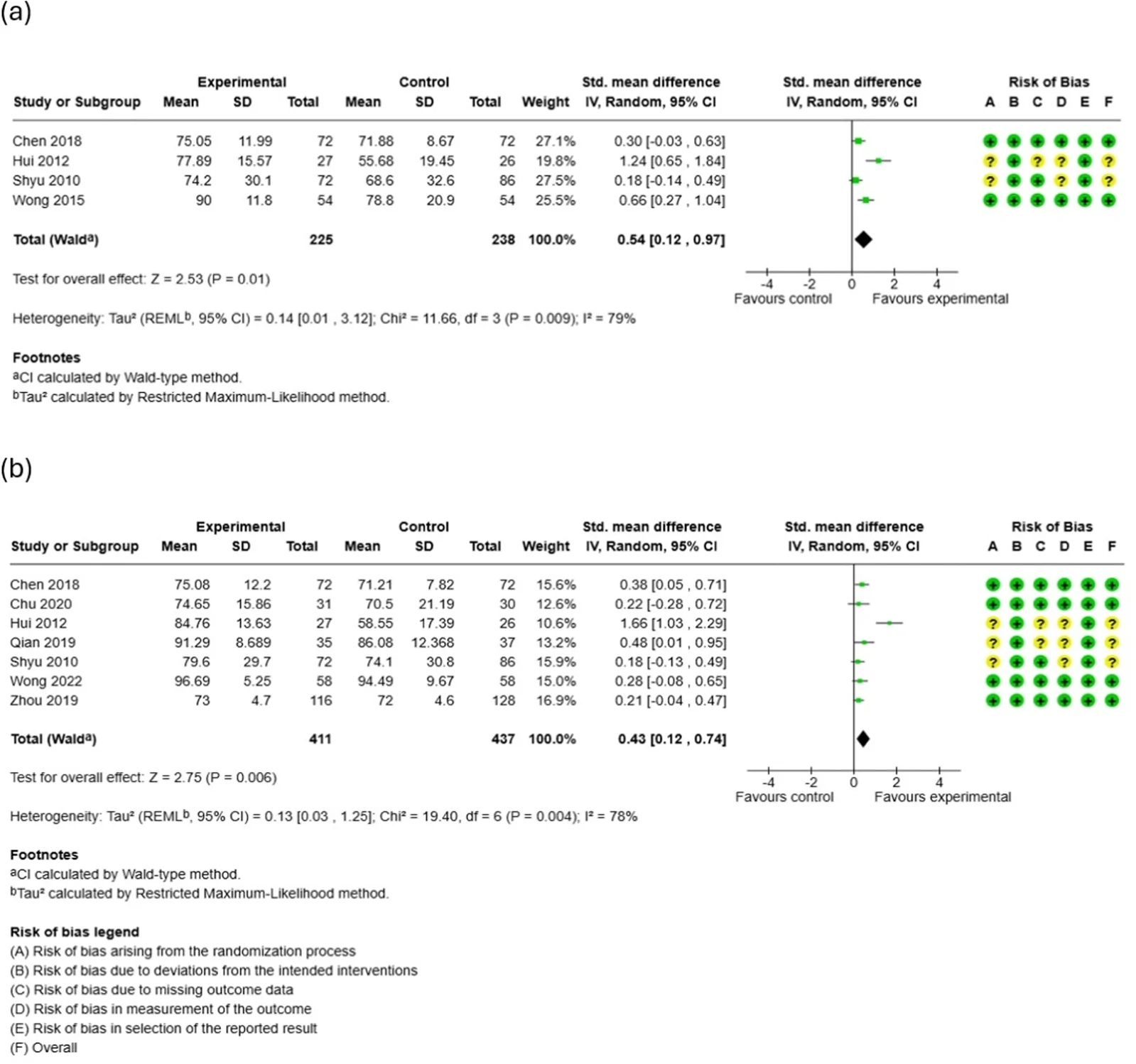
Forest plots for activities of daily living (ADLs) at (a) 1 month and (b) 3 months.

*Quality of life:* Quality of life was measured in 11 studies. Using the SF-36, the pooled effect sizes for quality of life subdomains showed significant between-group differences in the Mental Component Score at 1 month^25,38^ (RE, MD 0.73, 95% CI 0.34 to 1.12, I^2^ = 0%, 2 trials, 252 participants) (Figure 4). The certainty of the evidence was rated as low due to imprecision (Supplemental Table 7). There were no significant differences for the Mental Component Score at 3 months^25,38^ (RE, MD 0.88, 95% CI −0.50 to 2.25, I^2^ = 41%, 2 trials, 252 participants) (Supplemental Figures 2). No differences were observed in the Physical Component Score at either 1 or 3 months^25,38^ (Supplemental Figures 3 and 4). At 6 months, pooled results showed a favourable effect for the intervention group compared with the control group in the Role Limitations due to Physical Problems component score^45,47,49^ (RE, MD 7.70, 95% CI 0.17 to 15.23, I^2^ = 20%, 3 trials, 258 participants) and the General Health component score^45,47,49^ (RE, MD 5.32, 95% CI 0.87 to 9.77, I^2^ = 0%, 3 trials, 258 participants) (Figure 5). The certainty of the evidence was rated as very low due to risk of bias and imprecision (Supplemental Table 7). No significant between-group differences were found for the remaining six subdomains of the SF-36^45,47,49^ (Supplemental Figures 5–10). Six studies not included in the meta-analysis considered this outcome. As the overall quality of life score, one showed significant improvements in 1 month and 3 months (*p* < 0.001).^44^ Two showed significant improvements in quality of life at 3 months and 6 months.^27,28^ Three studies found no effect on quality of life at 6 months.^39–41^

**Figure 4.**
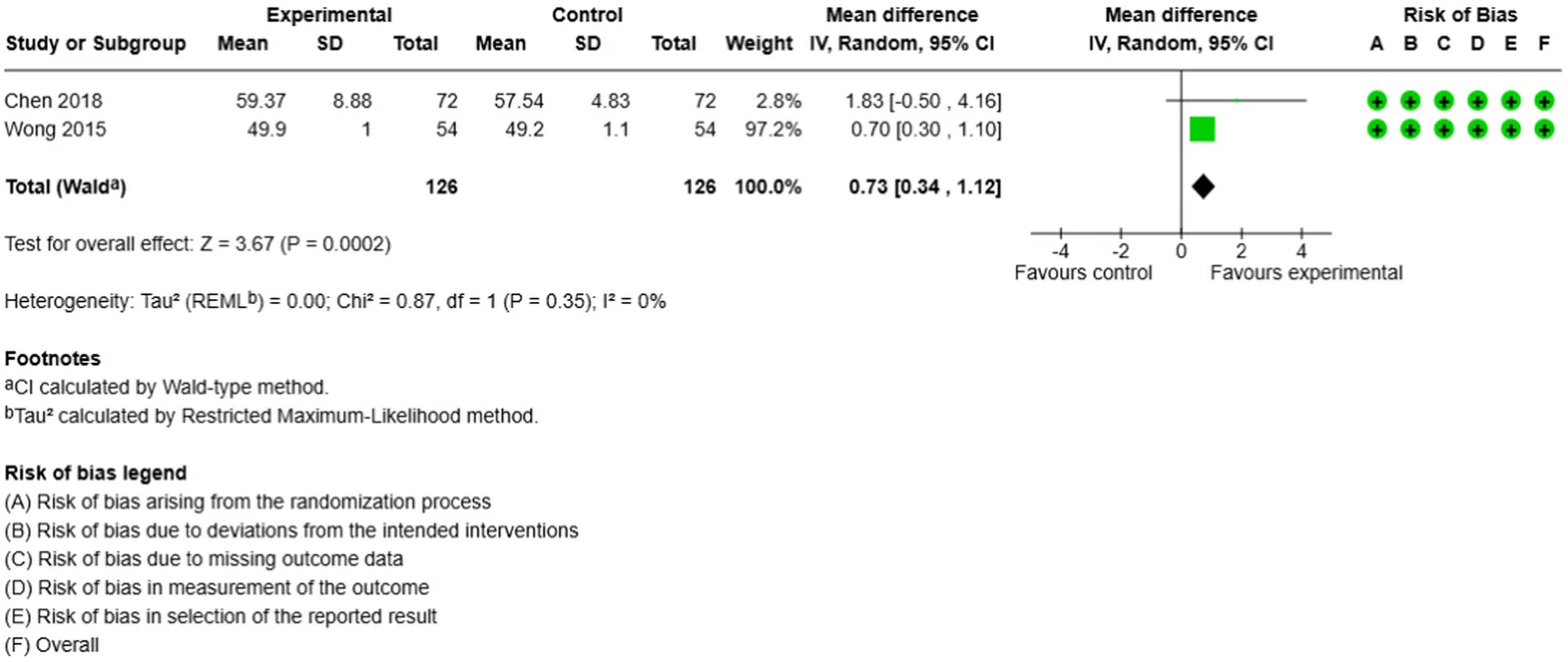
Forest plots for the mental component score (MCS) at 1 month.

**Figure 5.**
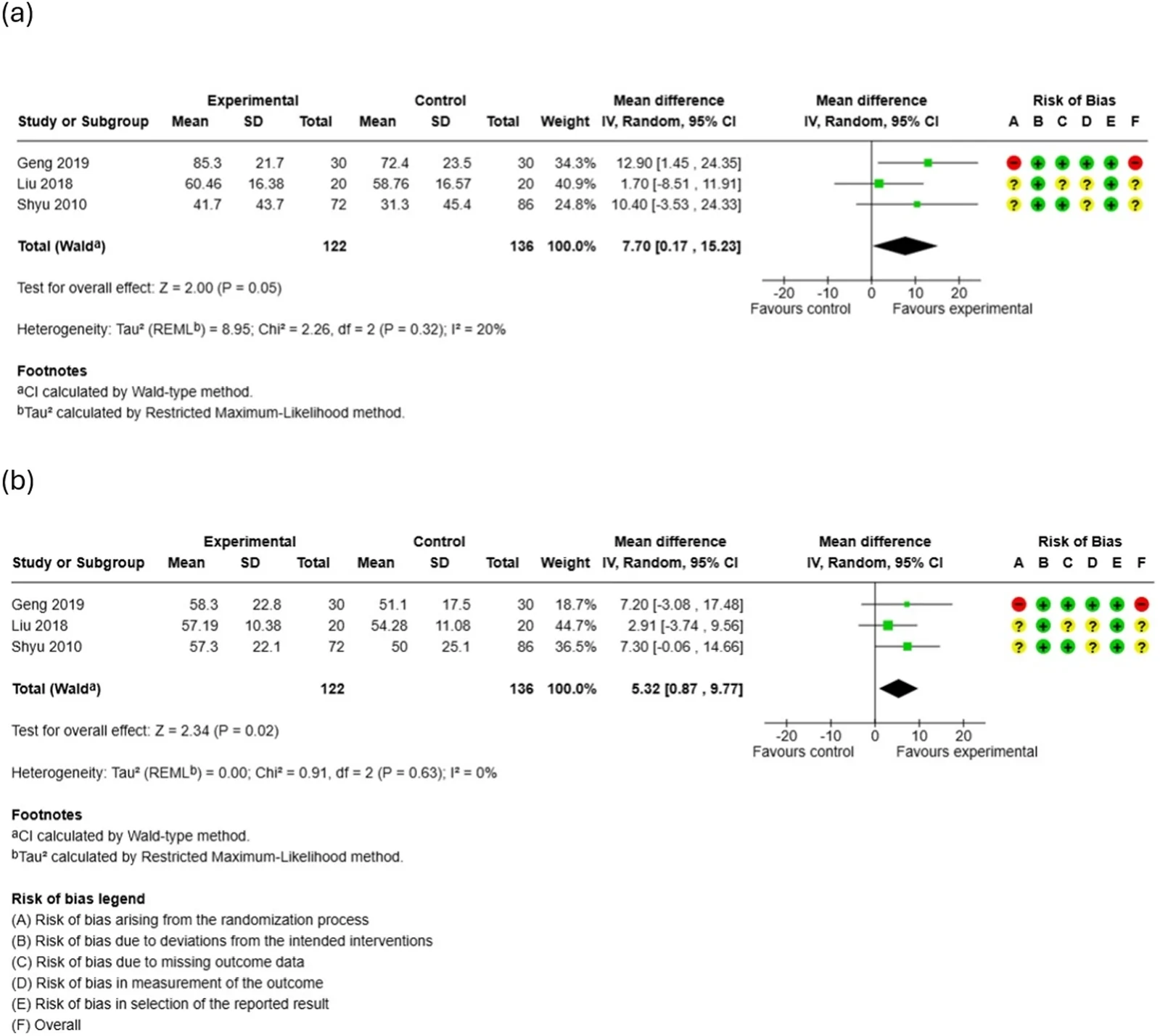
Forest plots for (a) the role limitations due to physical problems (RP) at 6 months and (b) the general health component (GH) at 6 months.

*Depression and anxiety*: Depression was measured in three studies. Meta-analysis of two studies showed no effect in favour of transitional care interventions on depression at 1 month^25,38^ (RE, SMD −1.08, 95% CI −2.19 to 0.03, I^2^ = 94%) (Supplemental Figure 11). A cluster-RCT^40^ found no important between-group differences in depression at 6 months (MD 0.1, 95% CI −0.5 to 0.7) and 12 months (MD −0.4, 95% CI −1.1 to 0.3), or in anxiety at 6 months (MD 0.1, 95% CI −0.5 to 0.7) and 12 months (MD −0.2, 95% CI −0.9 to 0.3).

*Self-efficacy*: Self-efficacy was measured in four studies. Pooled results indicated no effect in favour of transitional care interventions on self-efficacy at 3 months^27,28,38^ (RE, SMD 6.95, 95% CI −5.86 to 19.76, I^2^ = 100%) and 6 months^27,28^ (RE, SMD 9.84, 95% CI −8.73 to 28.42, I^2^ = 100%) (Supplemental Figures 12 and 13). One study^42^ not included in the meta-analysis considered this outcome and showed a statistically significant improvement in self-efficacy scores at 6 weeks (*p* < 0.001) and 18 weeks (*p* < 0.001).

*Stroke-related knowledge*: Stroke-related knowledge was measured in two studies. Meta-analysis showed no effect in favour of transitional care interventions on stroke knowledge at 3 months (RE, SMD 15.73, 95% CI −13.20 to 44.66, I^2^ = 100%)^27,47^ and 6 months (RE, SMD 14.41, 95% CI −11.67 to 40.50, I^2^ = 100%)^27,47^ (Supplemental Figures 14 and 15).

*Patient satisfaction*: Two studies reported the effect of the intervention on patient satisfaction with care.^25,48^ Meta-analysis indicated an effect in favour of transitional care interventions up to 3 months (RE, SMD 3.62; 95% CI 1.08 to 6.17, I^2^ = 96%, 2 trials, 180 participants; very low certainty of evidence (downgraded for inconsistency and imprecision)), effect was sustained in sensitivity analysis excluding studies with some concern (Supplemental Figure 16, Figure S7, Table 7).

*Caregiver burden:* Caregiver burden was measured in five studies. Pooled results of three studies^27,39,41^ reported no effect in favour of transitional care interventions on caregiver burden at 6 months (RE, SMD −6.77, 95% CI −20.02 to 6.47, I^2^ = 100%) (Supplemental Figure 17). Two studies^26,40^ not included in the meta-analysis considered this outcome. One showed significant differences between groups at two weeks post-discharge (*p* < 0.001),^26^ and the other showed no significant between-group differences at 6 months (MD 0.5, 95% CI −1.7 to 2.7).^40^

*Caregiver quality of life, self-efficacy, satisfaction:* Caregiver self-efficacy and satisfaction with care were not considered in the included studies, and only two studies^40,49^ evaluated caregivers’ quality of life. One study found no significant difference between the two groups at 1, 3, 6 and 12 months.^49^ A cluster-RCT^40^ found no significant improvement in caregivers’ quality of life at 6 months (MD −0.014, 95% CI −0.044 to 0.016) and 12 months (MD 0.019, 95% CI −0.013 to 0.050).

*Healthcare utilisation:* Six studies evaluated the effect of the intervention on hospital readmission, while three studies examined the effect on emergency department visits. The meta-analysis results indicated an effect in favour of transitional care interventions on hospital readmission up to 3 months^25,38,48^ (RE, RR 0.54; 95% CI 0.30 to 0.97; I^2^ = 0%, 3 trials, 324 participants) (Figure 6). The effect was sustained in sensitivity analysis excluding studies with high risk of bias (Supplemental Figure S9). The certainty of the evidence was rated as very low due to risk of bias and imprecision (Supplemental Table 7). There were no between-group differences in hospital readmission at 6 months^27,49^ (RE, RR 0.50, 95% CI 0.21 to 1.18, I^2^ = 52%) or in emergency department visits up to 3 months^25,48^ (RE, RR 0.42, 95% CI 0.09 to 2.00, I^2^ = 50%) (Supplemental Figures 18 and 19). One study^50^ not included in the meta-analysis considered this outcome and showed statistically significant differences in emergency department visits at 6 months.

**Figure 6.**
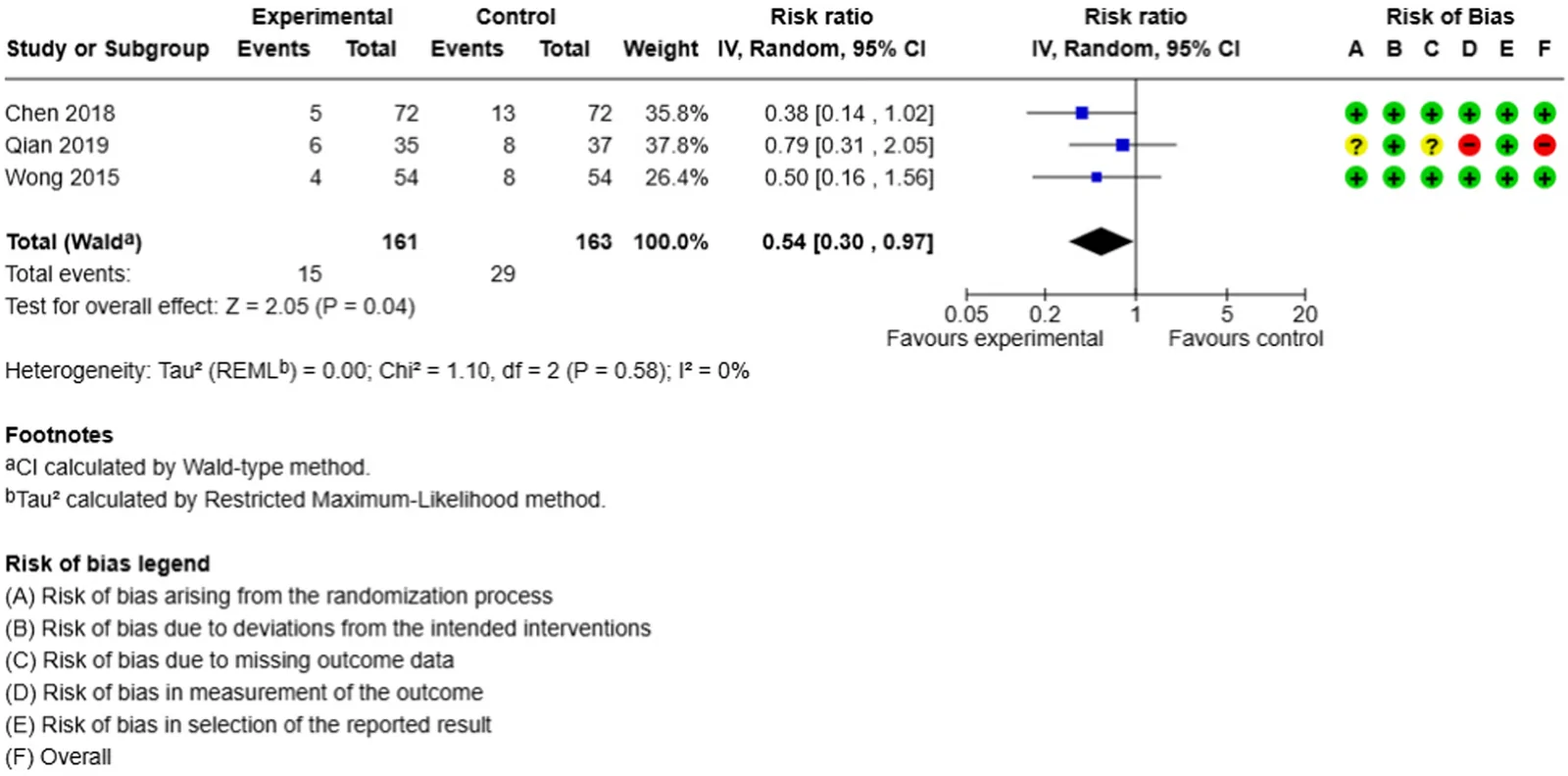
Forest plot for hospital readmissions up to 3 months.

*Subgroup analysis:* The post-hoc subgroup analyses are presented in Supplemental Figures G1.1 to G2.3. We found differences in the effects on activities of daily living in the subgroup analyses by contact mode and number of intervention components. The pooled SMDs of activities of daily living in the trials using home visits or phone calls alone and those using a combination of home visits and phone calls were 0.53 (95% CI −0.20 to 1.26) and 0.30 (95% CI 0.08 to 0.51), respectively. The pooled SMDs in the trials applied 2–3 components, and 4–5 components were 0.47 (95% CI −0.09 to 1.04) and 0.35 (95% CI 0.10 to 0.59), respectively. We found no difference in the effect of the findings across subgroup analyses on hospital readmissions.

## Discussion

Our meta-analysis shows that nurse-initiated transitional care with pre- and post-discharge components improved activities of daily living at 1 and 3 months. It also improved the Mental Component Score of quality of life at 1 month and the Role Limitations due to Physical Problems and General Health domains at 6 months post-discharge. These interventions enhanced patient satisfaction and reduced 3-month hospital readmissions. Sensitivity analysis excluding studies with some concerns or a high risk of bias showed stable effects on daily activities, satisfaction and readmissions. The certainty of evidence was low to very low. There were no significant effects on depression, anxiety, self-efficacy, stroke knowledge, emergency visits or caregiver burden. A meta-analysis of caregivers’ quality of life, self-efficacy and satisfaction was not possible due to limited data.

Although nurse-initiated transitional care interventions improved short-term activities of daily living in older stroke survivors, these benefits diminished once the intervention ended, consistent with prior studies showing gains at 3 months that are not sustained at 6–12 months.^16,17^ The reduced long-term effects observed in this review may be due to limited self-management or poor adherence following the intervention. Our subgroup analysis found multi-component transitional care produced greater three-month improvements, with home visits plus phone calls outperforming single approaches. This moderate effect indicates meaningful benefits for physical recovery in older stroke survivors.

The effects of transitional care on the subdomains of quality of life in older stroke survivors after discharge contrast with those of O’Callaghan et al., who found improvements in the Physical Component Score at 3 months, but no effects on the Mental Component Score or either subdomain of the SF-36 at 6–12 months in adult stroke survivors.^17^ Lin et al. reported positive effects of transitional care on the overall quality-of-life score at 3 months post-discharge.^16^ According to Bandura's self-efficacy theory, persistence in activities can be produced through mastery experiences, and perceived self-efficacy predicts performance.^52^ Despite self-management strategies maintaining self-efficacy and improving quality of life for both stroke survivors and caregivers,^38^ our review reveals that transitional care interventions primarily focus on health education rather than self-management support, which may lead to a lack of sustained improvement over time. In addition, most interventions were designed without input from stroke survivors or family members.^25,38–40^^,42–50^ Engaging key stakeholders in designing an intervention is therefore crucial for understanding the problem, prioritising needs and developing solutions for long-term self-efficacy in real-world settings.^53^

The findings on the effects of depression and anxiety in our review contrast with previous reviews, which reported reductions in depression at 3 months post-discharge^16^ and in both anxiety and depression between 6 and 12 months.^17^ Depression in older stroke survivors relates to low self-esteem, limited social support and reduced functional ability.^54^ Nurses and health professionals should recognise the risk and severity of depression and anxiety in this group, routinely screen, and provide emotional support and coping skills.^55^ Caregivers play a vital role in supporting older stroke survivors’ physical and psychological health as they return home.^6–8^ It is necessary to involve caregivers in transitional care and provide support to help them cope with their caregiving burden.^26^

This review's strength lies in its robust methodology, aligned with PRISMA guidelines.^29^ Several limitations should be noted. It is possible that some literature was missed due to the language and date limitations. The scope of this study was intended to encompass complex nursing transitional care interventions, leading to a broad search strategy that yielded a very large number of records, despite the language and date filters. We need to be cautious about analysis reporting bias, as most included studies did not publish a study protocol, limiting comparisons of changes in pre-specified outcomes. Variability across intervention characteristics and heterogeneity in outcome measurement made estimating the true effect size for each outcome challenging. Random-effects models were used to estimate pooled intervention effects and to address limitations in effect-size homogeneity, and heterogeneity was explored through post hoc subgroup analyses.^35^ The pooled effects are limited to associations observed across studies and cannot identify potential causal mechanisms, as inferences are made about the effectiveness of transitional care interventions as a whole, rather than for specific programmes or components. The overall certainty of the evidence was low to very low, due to high risk of bias or concerns, small sample sizes and high heterogeneity. The included studies were predominantly conducted in East Asia (China) and the Middle East (Iran), with only two originating in Europe and North America. The transferability of the findings of this study, or the adaptation of interventions for a new setting, needs to be considered in terms of intervention-context fit with healthcare systems, cultural contexts and target population.^56^

In conclusion, nurses play a central role in transitional (hospital-to-home) care, as intervention providers, case managers and team collaborators, supporting post-discharge recovery in older stroke survivors.^27,28,38,39,41,43–45^^,47–49^ Effective nurse-led transitional care should incorporate patient- and caregiver-centred assessment, goal setting, personalised care planning, stroke-specific education, self-management support, rehabilitation and a combination of home visits and phone calls to optimise outcomes. However, evidence is largely limited to short-term effects. Future research should confirm these findings, examine intervention intensity and duration, and incorporate the perspectives of survivors, caregivers and multidisciplinary teams to strengthen long-term effectiveness.

**Clinical messages**

- Nurse-led hospital-to-home programs may boost short-term effects on activities of daily living, quality of life and satisfaction, and reduce readmissions – though current evidence remains low-certainty.
- Multicomponent models (including home visits, follow-up calls and self-management support) show the most promise for enhancing activities of daily living recovery over time.
- Co-design of transitional care interventions with diverse stakeholders, including people with lived experience, is essential to support translation into practice.

## Supplemental Material

sj-docx-1-cre-10.1177_02692155261430092 - Supplemental material for Nurse-initiated transitional care interventions to support the transition from hospital to home amongst older stroke survivors and caregivers: A systematic review and meta-analysis Supplemental material, sj-docx-1-cre-10.1177_02692155261430092 for Nurse-initiated transitional care interventions to support the transition from hospital to home amongst older stroke survivors and caregivers: A systematic review and meta-analysis by Usanee Ankanawin, Lisa Kidd and Dawn A Skelton in Clinical Rehabilitation

## References

[1] Feigin VL Abdollahi A Abreu LG , et al. Global, regional, and national burden of stroke and its risk factors, 1990–2021: a systematic analysis for the global burden of disease study 2021. Lancet Neurol 2024; 23: 973–1003. 10.1016/S1474-4422(24)00369-7 PMC12254192 39304265

[2] Feigin VL Brainin M Norrving B , et al. World Stroke Organization: Global Stroke Fact Sheet 2025. Int J Stroke 2025; 20: 132–144. 10.1177/17474930241308142 PMC11786524 39635884

[3] World Health Organization. UN decade of healthy ageing plan of action (2021–2030), https://www.who.int/initiatives/decade-of-healthy-ageing (2021, accessed September 5, 2023).

[4] Predebon ML Pizzol FLFD Santos NO , et al. The capacity of informal caregivers in the rehabilitation of older people after a stroke. Inves Educ Enferm 2021; 39: e03. 10.17533/udea.iee.v39n2e03 PMC8253530 34214280

[5] Rodrigues RAP Marques S Kusumota L , et al. Transition of care for the elderly after cerebrovascular accidents - from hospital to the home. Rev Lat Am 2013; 21: 216–224. 10.1590/s0104-11692013000700027 23459910

[6] Camak DJ . Addressing the burden of stroke caregivers: a literature review. J Clin Nurs 2015; 24: 2376–2382. 10.1111/jocn.12884 26095074

[7] Min K Min J . Health-related quality of life is associated with stroke deficits in older adults. Age Ageing 2015; 44: 700–704. 10.1093/ageing/afv060 25994186

[8] Ofori-Asenso R Zomer E Chin KL , et al. Effect of comorbidity assessed by the Charlson comorbidity index on the length of stay, costs and mortality among older adults hospitalised for acute stroke. Int J Environ Res Public Health 2018; 15: 2532. 10.3390/ijerph15112532 PMC6267000 30424531

[9] Kuptniratsaikul V Kovindha A Suethanapornkul S , et al. Long-term morbidities in stroke survivors: a prospective multicenter study of Thai stroke rehabilitation registry. BMC Geriatr 2013; 13: Article33. 10.1186/1471-2318-13-33 PMC3635998 23586971

[10] Suri MF Qureshi AI . Readmission within 1 month of discharge among patients with acute ischemic stroke: results of the University HealthSystem Consortium Stroke Benchmarking study. J Vasc Interv Neurol 2013; 6: 47–51. PMC3868247 24358417

[11] Man S Bruckman D Tang AS , et al. The association of socioeconomic status and discharge destination with 30-day readmission after ischemic stroke. J Stroke Cerebrovasc Dis 2021; 30: 106146. 10.1016/j.jstrokecerebrovasdis.2021.106146 34644664

[12] Fonarow GC Smith EE Reeves MJ , et al. Hospital-level variation in mortality and rehospitalization for medicare beneficiaries with acute ischemic stroke. Stroke 2011; 42: 459–466. 10.1161/STROKEAHA.110.601831 21164109

[13] Lin S Wang C Wang Q , et al. The experience of stroke survivors and caregivers during hospital-to-home transitional care: a qualitative longitudinal study. Int J Nurs Stud 2022; 130: 104213. 10.1016/j.ijnurstu.2022.104213 35378465

[14] Freaney S . More planning and slower discharge: a qualitative study of the facilitators of and barriers to a smooth discharge for stroke patients. Br J Neurosci Nurs 2021; 17: S8.

[15] Coleman EA Boult C . Improving the quality of transitional care for persons with complex care needs. J Am Geriatr Soc 2003; 51: 556–557. 10.1046/j.1532-5415.2003.51186.x 12657079

[16] Lin S Xiao LD Chamberlain D , et al. The effect of transition care interventions incorporating health coaching strategies for stroke survivors: a systematic review and meta-analysis. Patient Educ Couns 2020; 103: 2039–2060. 10.1016/j.pec.2020.05.006 32532632

[17] O’Callaghan G Fahy M Murphy P , et al. Effectiveness of interventions to support the transition home after acute stroke: a systematic review and meta-analysis. BMC Health Serv Res 2022; 22: 1095. 10.1186/s12913-022-08473-6 PMC9420257 36031608

[18] Saragih ID Everard G Saragih IS , et al. The beneficial effects of transitional care for patients with stroke: a meta-analysis. J Adv Nurs 2023; 80: 789–806. 10.1111/jan.15850 37727124

[19] Wang Y Yang F Shi H , et al. What type of transitional care effectively reduced mortality and improved ADL of stroke patients? A meta-analysis. Int J Environ Res Public Health 2017; 14: 510. 10.3390/ijerph14050510 PMC5451961 28489044

[20] Bhalla A Clark L Fisher R , et al. The new national clinical guideline for stroke: an opportunity to transform stroke care. Clin Med 2024; 24: 100025. 10.1016/j.clinme.2024.100025 PMC11091392 38387534

[21] Mountain A Patrice Lindsay M Teasell R , et al. Canadian Stroke best practice recommendations: rehabilitation, recovery, and community participation following stroke. Part two: transitions and community participation following stroke. Int J Stroke 2020; 15: 789–806. 10.1177/1747493019897847 31983292

[22] Stroke Foundation. Clinical Guidelines for Stroke Management. Chapter 7 of 8: discharge planning and transfer of care (updated 2025 April), https://informme.org.au/en/Guidelines/Clinical-Guidelines-for-Stroke-Management (2025, accessed May 5, 2025).

[23] Rennke S Ranji SR . Transitional care strategies from hospital to home: a review for the neurohospitalist. Neurohospitalist 2015; 5: 35–42. 10.1177/1941874414540683 PMC4272352 25553228

[24] Camicia M Lutz B Summers D , et al. Nursing's role in successful stroke care transitions across the continuum: from acute care into the community. Stroke 2021; 52: e794–e805. 10.1161/STROKEAHA.121.033938 34727736

[25] Wong FKY Yeung SM . Effects of a 4-week transitional care programme for discharged stroke survivors in Hong Kong: a randomised controlled trial. Health Soc Care Community 2015; 23: 619–631. 10.1111/hsc.12177 25470529

[26] Farahani MA Ghezeljeh TN Haghani S , et al. The effect of a supportive home care program on caregiver burden with stroke patients in Iran: an experimental study. BMC Health Serv Res 2021; 21: 346. 10.1186/s12913-021-06340-4 PMC8048267 33858400

[27] Lin S Xiao LD Chamberlain D , et al. Nurse-led health coaching programme to improve hospital-to-home transitional care for stroke survivors: a randomised controlled trial. Patient Educ Couns 2022; 105: 917–925. 10.1016/j.pec.2021.07.020 34294494

[28] Wong FKY Wang SL Ng SSM , et al. Effects of a transitional home-based care program for stroke survivors in Harbin, China: a randomized controlled trial. Age Ageing 2022; 51: 1–10. 10.1093/ageing/afac027 35180283

[29] Page MJ McKenzie JE Bossuyt PM , et al. The PRISMA 2020 statement: an updated guideline for reporting systematic reviews. BMJ 2021; 372: n71. 10.1136/bmj.n71 PMC8005924 33782057

[30] Amir-Behghadami M Janati A . Population, intervention, comparison, outcomes and study (PICOS) design as a framework to formulate eligibility criteria in systematic reviews. Emerg Med J 2020; 37: 387. 10.1136/emermed-2020-209567 32253195

[31] Hoffmann TC Glasziou PP Boutron I , et al. Better reporting of interventions: template for intervention description and replication (TIDieR) checklist and guide. BMJ 2014; 348: g1687. 10.1136/bmj.g1687 24609605

[32] Sterne JAC Savović J Page MJ , et al. Rob 2: a revised tool for assessing risk of bias in randomised trials. BMJ 2019; 366: l4898. 10.1136/bmj.l4898 31462531

[33] Eldridge S Campbell MK Campbell MJ , et al. Revised cochrane risk of bias tool for randomized trials (RoB 2): additional considerations for cluster-randomized trials (RoB 2 CRT). *Cochrane*, 2021. https://www.riskofbias.info/welcome/rob-2-0-tool/rob-2-for-cluster-randomized-trials (2021, accessed April 10, 2025).

[34] Guyatt G Oxman AD Akl EA , et al. GRADE Guidelines: 1. Introduction—GRADE evidence profiles and summary of findings tables. J Clin Epidemiol 2011; 64: 383–394. 10.1016/j.jclinepi.2010.04.026 21195583

[35] Deeks JJ Higgins JP Altman DG , et al. Chapter 10: analysing data and undertaking meta-analyses. In: Higgins JP Thomas J Chandler J , et al. (eds) Cochrane handbook for systematic reviews of interventions (version 6.5). Cochrane, 2024. https://www.cochrane.org/authors/handbooks-and-manuals/handbook/current/chapter-10 (2024, accessed May 9, 2025).

[36] Higgins JP Li T Deeks JJ. Chapter 6: choosing effect measures and computing estimates of effect. In: Higgins JP Thomas J Chandler J , et al. (eds) Cochrane handbook for systematic reviews of interventions (version 6.5). Cochrane, 2024. https://www.cochrane.org/authors/handbooks-and-manuals/handbook/current/chapter-06 (2024, accessed May 9, 2025).

[37] Page MJ Higgins JP Sterne JAC , et al. Chapter 13: assessing risk of bias due to missing evidence in a meta-analysis. In: Higgins JP Thomas J Chandler J , et al. (eds) Cochrane handbook for systematic reviews of interventions (version 6.5.). Cochrane, 2024. https://www.cochrane.org/authors/handbooks-and-manuals/handbook/current/chapter-13 (2024, accessed May 9, 2025).

[38] Chen L Chen Y Chen X , et al. Longitudinal study of effectiveness of a patient-centered self-management empowerment intervention during predischarge planning on stroke survivors. Worldviews Evid Based Nurs 2018; 15: 197–205. 10.1111/wvn.12295 29878691

[39] Chu K Bu X Sun Z , et al. Feasibility of a nurse-trained, family member-delivered rehabilitation model for disabled stroke patients in rural Chongqing, China. J Stroke Cerebrovasc Dis 2020; 29: 105382. 10.1016/j.jstrokecerebrovasdis.2020.105382 33096497

[40] Forster A Dickerson J Young J , et al. A structured training programme for caregivers of inpatients after stroke (TRACS): a cluster randomised controlled trial and cost-effectiveness analysis. Lancet 2013; 382: 2069–2076. 10.1016/S0140-6736(13)61603-7 24054816

[41] Zhou B Zhang J Zhao Y , et al. Caregiver-delivered stroke rehabilitation in rural China the RECOVER randomized controlled trial. Stroke 2019; 50: 1825–1830. 10.1161/STROKEAHA.118.021558 31177978

[42] Amiri FS Abolhassani S Alimohammadi N , et al. Investigating the effect of self-management program on stroke's patients’ self-efficacy. BMC Neurol 2022; 22: 1–10. 10.1186/s12883-022-02876-y PMC9492461 36138361

[43] Hui X Chunhui Z Beilei L , et al. Influence of home visits nursing on activities of daily living in stroke patients. Life Sci J 2012; 9: 1583–1586.

[44] Jafari J Kermansaravi F Yaghoubinia F . The effect of home-based rehabilitation on adherence to treatment and quality of life of individuals after stroke. Med Surg Nurs J 2020; 9: e107716.

[45] Liu L . Effects of transitional care on functional exercise: compliance and health status of stroke patients. Acta Med Mediterr 2018; 34: 959–965.

[46] Torres-Arreola LP Doubova SV Hernandez SF , et al. Effectiveness of two rehabilitation strategies provided by nurses for stroke patients in Mexico. J Clin Nurs 2009; 18: 2993–3002. 10.1111/j.1365-2702.2009.02862.x 19821873

[47] Geng G He W Ding L , et al. Impact of transitional care for discharged elderly stroke patients in China: an application of the integrated behavioral model. Top Stroke Rehabil 2019; 26: 621–629. 10.1080/10749357.2019.1647650 31369355

[48] Qian CR Zhong DQ Shen YY , et al. Evaluation of clinical efficacy of transitional care mode for patients with strokes. Int J Clin Exp Med 2019; 12: 981–988.

[49] Shyu YIL Kuo LM Chen MC , et al. A clinical trial of an individualised intervention programme for family caregivers of older stroke victims in Taiwan. J Clin Nurs 2010; 19: 1675–1685. 10.1111/j.1365-2702.2009.03124.x 20579205

[50] Wong AKC Wang SL So C , et al. Economic evaluation of an enhanced post-discharge home-based care program for stroke survivors. Value Health 2024; 27: 405–414. 10.1016/j.jval.2024.01.012 38309655

[51] Sawilowsky SS . New effect size rules of thumb. J Mod Appl Stat Methods 2009; 8: 597–599.

[52] Bandura A . Self-efficacy: toward a unifying theory of behavioral change. Psychol Rev 1977; 84: 191–215. 10.1037//0033-295x.84.2.191 847061

[53] Skivington K Matthews L Simpson SA , et al. A new framework for developing and evaluating complex interventions: update of Medical Research Council guidance. BMJ 2021; 374: n2061. 10.1136/bmj.n2061 PMC8482308 34593508

[54] Chau JP Thompson DR Chang AM , et al. Depression among Chinese stroke survivors six months after discharge from a rehabilitation hospital. J Clin Nurs 2010; 19: 3042. 10.1111/j.1365-2702.2010.03317.x 21040011

[55] Cameron JI O'Connell C Foley N , et al. Canadian Stroke best practice recommendations: managing transitions of care following stroke, guidelines update 2016. Int J Stroke 2016; 11: 807–822. 10.1177/1747493016660102 27443991

[56] Moore G Campbell M Copeland L , et al. Adapting interventions to new contexts—the ADAPT guidance. BMJ 2021; 374: n1679. 10.1136/bmj.n1679 PMC8329746 34344699

