# Supplementary material for: Nurse-initiated transitional care interventions to support the transition from hospital to home amongst older stroke survivors and caregivers: A systematic review and meta-analysis
Source: Clin Rehabil. 2026 Jun 10;40(11):1438–55. doi: 10.1177/02692155261430092 (PMC13736524; doi:10.1177/02692155261430092)
Supplement: Supplementary file 1 [file sj-docx-1-cre-10.1177_02692155261430092.docx]

**Supplemental material**

**Nurse-initiated transitional care interventions to support the transition from hospital to home amongst older stroke survivors and caregivers: A systematic review and meta-analysis**

Supplementary Table include:

Table 1. Protocol amendments

Table 2. Search strategy

Table 3. Characteristics of Included Studies

Table 4. Summary Risk of Bias of Included Studies

Table 5. Risk of Bias of Included Studies for each outcome

Table 6: Summary of shortlist of intervention components and underpinning theories

Table 7: GRADE summary of findings

Supplementary Figure include:

Forest Plot of Outcomes (Figure 1 – 19)

Sensitivity Analysis (Figure S1 – S9)

Subgroup Analysis (Figure G1.1 – G2.3)

**Table 1.** Protocol amendments

|  | Specified in protocol | Amendment | Justification |
| --- | --- | --- | --- |
| Population | Older stroke survivors, defined as individuals aged 60 years or older according to the World Health Organization, | Older stroke survivors—defined as individuals aged 60 years or older according to the World Health Organization^3^, or those with a mean age of ≥ 60 years in studies that included stroke survivors of any age. | Many studies did not specifically include stroke survivors aged 60 years and over; however, the majority of participants in these studies were older than 60. Therefore, we included studies having a mean age of stroke survivors ≥ 60 years in studies that included stroke survivors of any age. |
| Intervention | The duration of transitional care intervention encompasses the immediate transition  period, starting from the hospital and extending up to one month after discharge, reflecting the high risk of readmission  within 30 days after discharge for older stroke survivors. | The duration of transitional care intervention in this review encompasses both pre-discharge and post-discharge periods, lasting for up to six months after discharge. | There is variation in how transitional care interventions are defined across literature. Additionally, transitional care interventions for stroke survivors most commonly last 4 to 12 weeks, with the longest programme extending up to 24 weeks following discharge.^18^ Therefore, the duration of transitional care intervention in this review encompasses both pre-discharge and post-discharge periods, lasting for up to six months after discharge. This timeframe was selected to capture the impact of nurse-initiated transitional care on short term and long-term outcomes of older stroke survivors and caregivers. |

**Table 2. Search strategy**

Major search terms used as keywords and/or subject headings include stroke, transitional care/transition home/patient discharge, and nurses. The search strategy will include additional keywords, abbreviations, and MeSH terms as follows:

( stroke* OR "acute stroke*" OR "cerebrovascular accident*" OR "cerebrovascular stroke*" OR "cerebral stroke*" OR "stroke survivor*" OR CVA OR “cerebral infarction” ) AND ( "transition* care*" OR "transition* home" OR "transition* of care" OR "care* transition" OR “transition*” OR "hospital to home*" OR “discharge plan*” OR "patient discharge*" OR “hospital discharge*” OR discharge* OR "coach* program*" OR "health coach*" OR "caregiver-oriented" OR "holistic care" ) AND ( nurse* OR nursing OR "nurse-led" OR multidisciplinary OR integrated OR "nurse-coordinated" OR interprofessional* )

Interface: **EBSCOhost Research Database**

Search Screen: Advance Search

**Database: MEDLINE Search**

Published Date: 20090101-20240231

Search modes: Find all my search terms

Published Date: 20240301-20250304 **(updated search)**

Published Date: 20250304-20260303 **(updated search)**

Interface: **EBSCOhost Research Database**

Search Screen: Advance Search

**Database: CINAHL Complete (Cumulated Index for Nursing and Allied Health)**

Published Date: 20090101-20240231

Search modes: Find all my search terms

Published Date: 20240301-20250304 **(updated search)**

Published Date: 20250304-20260303 **(updated search)**

Interface: **ProQuest** **Research Database**

Search Screen: Advance Search

**Database: Health and Medical Collection Search**

Published Date: 20090101-20240229

Search modes: Find all my search terms

Published Date: 20240301-20250304 **(updated search)**

Published Date: 20250304-20260303 **(updated search)**

Interface: **ProQuest Research Database**

Search Screen: Advance Search

**Database: Nursing and Allied Health Search**

Published Date: 20090101-20240229

Search modes: Find all my search terms

Published Date: 20240301-20250304 **(updated search)**

Published Date: 20250304-20260303 **(updated search)**

Interface: **Web of Science Search**

Search Screen: Advance Search

**Database: Web of Science Search**

Published Date: 20090101-20240229

Search modes: Find all my search terms

Published Date: 20240301-20250304 **(updated search)**

Published Date: 20250304-20260303 **(updated search)**

Interface: **EBSCOhost Research Database**

Search Screen: Advance Search

**Database: Health Source: Nursing/Academic Edition Search**

Published Date: 20090101-20240231

Search modes: Find all my search terms

Published Date: 20240301-20250304 **(updated search)**

Published Date: 20250304-20260303 **(updated search)**

Interface: **SCOPUS Database**

Search Screen: Advance Search

**Database: SCOPUS Search**

Published Date: 20090101-20240229

Search modes: Find all my search terms

Published Date: 20240301-20250304 **(updated search)**

Published Date: 20250304-20260303 **(updated search)**

Interface: **CENTRAL**

Search Screen: Advance Search

**Database: the Cochrane Central Register of Controlled Trials (CENTRAL) Search**

Published Date: 20090101-20240224

Search modes: Find all my search terms

Published Date: 20240301-20250304 **(updated search)**

Published Date: 20250304-20260303 **(updated search)**

**Table 3**. **Characteristics of Included Studies**

| **First author, year, country**; **Design** | **Sample size (N),**  **Age (year, mean, SD)**  **Type of Stroke (n, %)**  **Recruitment setting** | **Intervention characteristics** | | | | | **Outcomes** |
| --- | --- | --- | --- | --- | --- | --- | --- |
|  |  | **Intervention name and provider** | **Intervention components** | **Control group** | **Intervention length**  **and Frequency (weeks or months)** | **Follows-up length**  **(week or month)** | **Outcome Measures (Scale)** |
| Amiri 2022, Iran, RCT | **N = 72 stroke survivors**  IG = 36, mean age 69.1 (SD 8.64) yrs  CG = 36, mean age 67.67 (SD 8.30) yrs  **Type of Stroke (n, %):**  IG  Ischemic 35 (97.2%)  Hemorrhagic 1 (2.8%)  CG  Ischemic 36 (100%)  Hemorrhagic 0 (0%)  **Recruitment setting:**  Neurology departments | 5A self-management model  **Provider**  Nurse researcher, Psychiatric nurse, Physiotherapist | **Predischarge**  *The first and second stage: assessment and advice*  Bedside needs assessment in knowledge, attitude and practice (e.g., completion of questionnaire, relapse prevention, benefits of behavior change), develop educational contents.  **Post discharge**  *The third stage: agree*  set and accomplish behavioral goals  *The fourth stage: assist* virtual meetings for training (e.g., treatment conditions, medication / symptom / psychological management, lifestyle, social support, effective communication, problem-solving skills)  *The fifth stage: follow-up*  telephone follow-ups to answer questions.  **Materials**  - Educational booklet.  - Videos of educational cases | **Standard care**  **Predischarge**  Provide routine care during discharge  **Post discharge**  Telephone follow-up for 3 months. | 7 weeks  **Predischarge**  1 hour during discharge  **Post discharge**  7 weeks: virtual training from the third week to the sixth week,  telephone follow-ups in week 7. | Baseline,  After intervention,  3 months | Self-efficacy (SSEQ) |
| Chen 2018, China, RCT | **N = 144 stroke survivors**  IG = 72, mean age 65.92 (SD 12.80) yrs  CG= 72, mean age 64.78 (SD 9.87) yrs  **Type of Stroke (n, %):**  IG  Ischemic 52 (72.22%)  Hemorrhagic 20 (27.78%)  CG  Ischemic 55 (76.39%)  Hemorrhagic 17 (23.61%)  **Recruitment setting:**  Department of Neurology | Patient -centered self-management empowerment intervention (PCSMEI)  **Provider**  Nurse | **Predischarge**  - Patient and caregiver-centered assessment: patients health status, stroke knowledge, functional ability, worries, and rehabilitation goals.  - Five Face-to-face- daily sessions at bedside (e.g., functional status, stroke risk factors, problem-solving, complication preventions, functional rehabilitation, self-health monitoring strategies).  -Face-to-face small group sessions (stroke self-management, self-care skills, self-efficacy, self-management).  - Discharge assessment at bedside for setting rehabilitation and self-management goals.  **Post discharge**  Telephone follow-ups (patient’s performance, barriers/problems, problem-solving skills, goal accomplishment positive reinforcements.  **Materials**  - Workbook  - DVD | **Standard care**  **Predischarge**  Provide unstructured health education  **Post discharge**  Medical follow-up and telephone calls of general social chatting for 4 weeks. | **6 weeks**  **Predischarge**  - First week: assessment, five daily individual sessions (20 min per session)  - Second week; one small group session (60 min)  **Post discharge**  4 weeks: weekly telephone follow-ups (20-30 min sessions) | Baseline,  Discharge,  1 month,  3 months | ADLs (BI)  QoL (SF-36)  Depression (CES-D)  Self-efficacy (SSEQ)  Re-hospitalization |
| Chu 2020, China, RCT | **N = 61 stroke survivors**  IG = 31, mean age 62.84 (SD 9.71) yrs  CG = 30, mean age 66.17 (SD 8.51) yrs  **Type of Stroke (n, %):**  IG  Ischemic 17 (54.84%)  Hemorrhagic 14 (45.16%)  CG  Ischemic 19 (63.33%)  Hemorrhagic 11 (36.67%)  **Recruitment setting:**  Neurological wards | Nurse-Trained, Family Member-Delivered Rehabilitation Model  **Provider**  Nurse | **Predischarge**  - Develop a personized rehabilitation plan and provide rehabilitation skills training (mobility, self-care, and bowel/toilet control, etc.) for patients and caregivers  - Using teach- back techniques to assess the caregivers’ mastery of the training content.  **Post discharge**  - Caregivers delivered rehabilitation training  - Telephone follow-ups to monitor patient's progress and provide further guidance.  **Material**  - Rehabilitation instruction manuals | **Standard care**  **Predischarge**  Provide conventional care and rehabilitation  **Post discharge**  Post-hospital rehabilitation and follow- up. | **8 weeks**  **Predischarge**  Training once a day, 60 min each time, a total of 3 times  **Post discharge**  Telephone followed up in week 2, 4 and 8. | Baseline,  Pre-discharge,  3 months,  6 months | ADLs (BI)  QoL (EQ-5D)  Caregiver burden (CBI) |
| Farahani 2021, Iran, RCT | **N = 116 stroke survivors**  IG = 58, mean age 68.5 (SD 13.16) yrs  CG = 58, mean age 64.65 (SD 12.2) yrs  **N = 116 caregivers**  IG = 58, mean age 43.98 (SD 13.8) yrs  CG = 58, mean age 43.41 (SD 11.25) yrs  **Type of Stroke (n, %):**  Not reported  **Recruitment setting:**  Hospital wards | Supportive home care programme (aimed at carers)  **Provider**  Nurse | **Predischarge**  Two educational sessions: (1) support plan plus educational booklet.  (2) Knowledge about stroke, patient care, problem-solving, coping skills, communication with patients, active listening skills.  **Post discharge**  Home visits to provide six sessions: 3) Anger management, nutritional management, (4) Blood pressure control, PT of the limbs and respiratory system and pressure ulcer prevention, (5) Deep breathing exercises, hygiene care/suction, (6) Medication management, (7) Speech therapy and assessment of the needs for counseling with a psychologist, (8) Follow-up to assess for further referrals.  **Materials/Resources**  Educational booklet | **Standard care**  **Predischarge**  Provide routine education about CVA and caring patients with CVA | **2 weeks**  **Predischarge**  2 educational sessions after the stabilization of the  patient’s condition.  **Post discharge**  2 weeks home visit, each session lasts between 45 -60 min | Baseline,  2 weeks | Caregiver burden (CBI) |
| Forster 2013, UK,  Cluster RCT | **N = 928 stroke survivors**  IG = 450, mean age 71.0 (SD 12.76) yrs  CG = 478, mean age 71.3 (SD 12.18) yrs  **N = 928 caregivers**  IG = 450, mean age 61.1 (SD 14.64) yrs  CG = 478, mean age 60.8 (SD 13.91) yrs  **Type of Stroke** (n, %):  IG  Cerebral infarction 380 (84.4%)  Primary intracerebral hemorrhage 56 (12.4%)  Subarachnoid hemorrhage 6 (1.3%)  CG  Cerebral infarction 401 (83.9%)  Primary intracerebral hemorrhage 72(15.1%)  Subarachnoid hemorrhage 5 (1.0%)  **Recruitment setting:**  Stroke units | London Stroke Carers Training Course) Structured training  **Provider**  MDT (Physiotherapist, SALT, Nurse, HCA, Medical staff, social worker, Stroke association worker, Dietician) | **Predischarge**  14 structured training items  - 6 items mandatory, e.g., providing information on the nature of stroke and the importance of medication adherence.  - 8 items were based on individual caregiver needs, e.g., providing nutritional support for patients with swallowing difficulties.  **Post discharge**  One follow-up session after discharge via visit or phone call  **Materials/Resources**  Training manual | **Standard care**  **Predischarge**  - Provide information and advice based on National Clinical  Guidelines  - Set goals for rehabilitation and discharge  planning | **6 months**  **Predischarge**  At any time from admission, and throughout the patients stay  **Post discharge**  One follow-up session after discharge | Baseline,  6 months,  12months | Functional independence (NEADL, BI)  QoL (EQ-5D)  Stroke Impact Scale (SIS)  Anxiety (HADS-A)  Depression (HAD-S)  Caregiver burden (Caregivers burden scale)  Caregiver QoL (EQ-5D) |
| Geng 2019, China,  RCT | **N = 60 stroke survivors**  IG = 30, mean age 67.6 (SD 4.3) yrs  CG = 30, mean age 68.9 (SD 4.4) yrs  **Type of Stroke (n, %):**  IG  Ischemic 21 (70.0%)  Hemorrhagic 9 (30.0%)  CG  Ischemic 21 (70.0%)  Hemorrhagic 9 (30.0%)  **Recruitment setting:**  The Department of Neurology | Integrated  Behavioral Model (IBM) based  Transitional care  **Provider**  Neurological nurse practitioner/  TC nurses (from community health centers)/ neurologist,  rehabilitation counselor,  psychologist | **Predischarge**  - The neurologist and neurological nurse practitioner provided bedside health education with a learning booklet for patients and family caregivers.  **Post discharge**  - Home visit and telephone follow-up for patients’ and family caregivers’ needs assessment by the TC nurses and other TC team members follow-up if needed.  **Materials/Resources**  Learning booklet “Recovering after a stroke: a patient and family guide” | **Standard care**  **Predischarge** Bedside health education  **Post discharge**  Telephone follow-up one week after discharge and routine health checks at the hospital out-patient clinic after discharge. | **12 weeks**  **Predischarge**  30-min bedside health education  **Post discharge**  Weekly home visits, one day per week, and telephone follow-up one day per week for 12 weeks | Baseline,  1 month,  3 months,  6 months | ADLs (MBI)  QoL (SF-36)  Stroke knowledge (Stroke knowledge questionnaire) |
| Hui 2012, China, RCT | **N = 53 stroke survivors**  IG = 27  CG = 26  Mean age 63.41 (SD 11.08) yrs  **Type of Stroke (n, %):**  Ischemic 43 (81.13%)  Hemorrhagic 8 (15.09%)  Other 2 (3.78%)  **Recruitment setting:**  Neurology Departments | Home visits Nursing  **Provider**  Nurse | **Predischarge**  Hospital leaving instructions and home-visit plan  **Post discharge**  Home visit for health education (home environment, diet nutrients, and precautions in daily) and training of activities of daily living (e.g., how to dress, feeding, going toilet, instruction to use their affected hands to hold bowls, feeding with healthy hands)  **Materials/Resources**  Not reported | **Standard care**  **Predischarge**  Conventional hospital leaving instructions | **3 months**  **Predischarge**  1 discharge instruction  **Post discharge**  Home visits, once a week in the first month; once every two weeks  in the second month; and once in third month; 30-60  minutes at every turn | Baseline,  1 month,  3 months | ADLs (MBI) |
| Jafari 2020, Iran, RCT | **N = 60 stroke survivors**  IG = 30, mean age 60.70 (SD 7.33) yrs  CG = 30, mean age 60.20 (SD 4.35) yrs  **Type of Stroke (n, %):**  IG  Ischemic 30 (100 %)  CG  Ischemic 30 (100 %)  **Recruitment setting:**  The Internal  Medicine and Neurology Department | Home-Based Rehabilitation (HBR)  **Provider**  Nurse | **Predischarge**  Three sessions of knowledge about the disease and Home-Based Rehabilitation, and caregivers demonstrate knowledge apply and repeat the training.  (1) Stroke knowledge, rehabilitation regimen  (2) Diet education  (3) Medication regimen education  **Post discharge**  Phone calls or in-person conversations and caregivers call whenever needed  **Materials/Resources**  Educational booklet | **Standard care**  **Predischarge**  Provide usual  hospital instructions | **2 weeks**  **Predischarge**  Three 45-minute sessions after the acute phase (48-72 hours)  **Post discharge**  2 weeks: telephone call twice a week. | 1 month,  3 months | QoL (SS-QOL-49) |
| Lin 2022, China, RCT | **N = 140 stroke survivors**  IG = 70, mean age 60.8 (SD 11.0) yrs  CG = 70, mean age 62.5 (SD 10.9) yrs  **N = 140 caregivers**  IG = 70, mean age 54.5 (SD 11.9) yrs  CG = 70, mean age 53.8 (SD 11.0) yrs  **Type of Stroke (n, %):**  IG  Ischemic 66(94.3%)  Hemorrhagic 4(5.7%)  CG  Ischemic 65(92.9%)  Hemorrhagic 5(7.1%)  **Recruitment setting:**  Stroke wards | Nurse-led health coaching programme  **Provider**  Nurse | **Predischarge**  Five Individualized coaching sessions  1) Develop transitional care goals, personal action plan, and demonstrate self-care skills  2) Creating a safe home environment  3) Functional ability rehabilitation and exercise plan  4) Medication management  5) Complication prevention  **Post discharge**  - Weekly telephone support to discuss concern and problems.  - Bi-weekly face-to-face coaching activities at the outpatient department.  **Materials/Resources**  - Stroke Health Coaching Diary  - Education booklet | **Standard care**  **Predischarge**  Usual transitional discharge plan (verbal stroke-related education)  **Post discharge**  Telephone follow-ups at the end of the first and the fourth week. | **12 weeks**  **Predischarge**  5 coaching sessions (20-30 min each), three days prior to discharge  **Post discharge**  12 weeks: weekly telephone support (10-15 min each) and bi-weekly face-to-face coaching. | Baseline,  3 months,  6 months | QoL (SSQoL-12)  Self-efficacy (SSEQ)  Stroke-related health knowledge (SPKQ)  Unplanned hospital readmissions  Caregiver-related burden (Modified Caregiver Strain Index) |
| Liu 2018, China,  RCT | **N = 40 stroke survivors**  IG = 20, mean age 76.32 (SD 4.56) yrs  CG = 20, mean age 74.12 (SD 3.12) yrs  **Type of Stroke (n, %):**  Not reported  **Recruitment setting:**  Department of Neurology | Nurse-led transitional care intervention  **Provider**  Nurse, physician, rehabilitation therapist | **Predischarge**  - Patients and caregivers received discharge assessments (functional status, activities of daily living, drug use, cognition, attitude, and treatment beliefs about functional exercise, family, and social support)  - Developing a personalized transitional care plan based on the results of the assessment (health education and functional exercise program)  **Post discharge**  - 1 home visit for instruction patients and caregivers to perform functional exercises after discharge according to the exercise program  - Telephone follow-up for individual guidance  **Materials/Resources**  Exercise program brochure | **Standard care**  **Predischarge**  Provide routine instructions  on physical functional  exercise, daily care, dietary guidance, psychological  care, and medication guidance. | **4 weeks**  **Predischarge**  1 discharge instruction  **Post discharge**  1 home visit and telephone follow-up every day for 1 month | Baseline,  1 months,  3 months,  6 months | QoL (SF-36) |
| Qian 2019, China, RCT | **N = 72 stroke survivors**  IG = 35, mean age 66.54 (SD 10.16) yrs  CG = 37, mean age 64.8 (SD 10.49) yrs  **Type of Stroke (n, %):**  Not reported  **Recruitment setting:**  Hospital wards | Transitional nursing care mode  **Provider**  Nurse | **Predischarge**  A small-class health education curriculum and distribute self-designed diary for recording after discharge (e.g. rehabilitation training, diet adjustment, lifestyle, rational use of medication and regular follow-up).  **Post discharge**  - Telephone follow-ups for appropriate health instructions/counseling to screen risk factors of stroke, assistance regarding rehabilitation training methods and referrals to physicians and nurses, when necessary.  - Home visit for home rehabilitation exercises.  **Materials/Resources**  Self-designed diary | **Standard care**  (No detail about standard care) | **5 weeks**  **Predischarge**  1 week before discharge  **Post discharge**  Week 1: telephone follow-ups  Week 4: Home visits | 3 days before discharge,  3 months | ADLs (BI)  Patient satisfaction with nursing care (PSHC)  Frequency of hospitalization health service  Frequency of emergency health service |
| Shyu 2010, Taiwan, RCT | **N = 158 stroke survivors**  IG = 72, mean age 74.1 (SD 6.5) yrs  CG = 86, mean age 74.2 (SD 6.5) yrs  **N = 158 caregivers**  IG = 72  CG = 86  Overall age mean 47.9 (SD 13.7) yrs  **Type of Stroke (n, %):**  IG  Ischemic 22 (30.6%)  Infarction 41 (56.9%)  Hemorrhagic 8 (11.1%)  Transient ischemic attack 1 (1.4%)  CG  Ischemic 41 (47.7%)  Infarction 36 (41.9%)  Hemorrhagic 7 (8.2%)  Transient ischemic attack 2 (2.3%)  **Recruitment setting:**  Neurological wards | Family caregiver-oriented intervention programme  **Provider**  Research Nurses | **Predischarge**  - Assess individual discharge needs  - Consultation to anticipate competing needs after discharge  - Individualized discharge health educational consultation and referrals  **Post discharge**  Telephone follow-ups and home visits to detect/resolve problems or demonstrate skills  **Materials/Resources**  Not report | **Standard care**  **Predischarge**  Standard health education and consultation  **Post discharge**  Referrals when needed skilled home nursing service or nursing home placement | **4 weeks**  **Predischarge**  4-5 visits/ 30-min within 48 hours of admission  **Post discharge**  One 30–45-minute telephone call within the first week and two 30-minute home visits in week 1 and 4. | 1 month,  3 months,  6 months,  12 months | ADLs (BI)  QoL (SF-36),  Hospital readmission rate  Caregiver health-related QoL (SF-36) |
| Torres-Arreola, 2009, Mexico,  RCT | **N = 110 stroke survivors**  IG = 59, mean age 69.4 (SD 12.0) yrs  CG = 51, mean age 69.8 (SD 8.8) yrs  **Type of Stroke (n, %):**  Not reported  **Recruitment setting:**  Hospital wards | Nurse-directed rehabilitation programme  **Provider**  Nurse | **Predischarge**  The first strategy (S1)  - Education part (general care such as feeding, hygiene and mobilization) and a practical rehabilitation training session)  - Physiotherapy part (Intensive daily physical therapy)  **Post discharge**  - Daily physical therapy for 2 weeks,  - Bi-weekly in-home visit for 2 weeks,  - Weekly in-home visit for 3 months.  **Materials/Resources**  Manual of Physical Rehabilitation for the patient with Stroke | **The second strategy (S2 or** **standard care)**  **Predischarge**  Education part (general care such as feeding, hygiene and mobilization) and a practical rehabilitation training session)  **Post discharge**  8 home visits (45 min each) over 4 months | **4 months**  **Predischarge**  Daily education and physical therapy after 72 hours hospitalization  **Post discharge**  - Daily home visit for 2 weeks,  - Bi-weekly home visit for 2 weeks,  - Weekly home visit for 3 months. | Baseline,  1 month,  3 months,  6 months | ADLs (BI) |
| Wong 2015, China, RCT | **N = 108 stroke survivors**  IG = 54, mean age 67.5 (SD 11.6) yrs  CG = 54, mean age 71.5 (SD 11.6) yrs  **Type of Stroke (n, %):**  IG  Ischemic 47 (87.0%)  Hemorrhagic 7 (13.0%)  CG  Ischemic 47 (87.0%)  Hemorrhagic 7 (13.0%)  **Recruitment setting:**  Hospital wards | Transitional care programme (TCP)  **Provider**  Holistic care managers (nurse) | **Predischarge**  Family meeting, pre-discharge holistic assessment, mutual goals setting, care planning based on four domains of the Omaha system (environmental, psychosocial, physiological and health-related behavior) and six domains of clinical guidelines for stroke patients (management and prevention of stroke recurrence, symptom management, self-care ability and exercise, healthy behavior, adherence to medication and diet, building resilience, emotional management)  **Post discharge**  Home visit and telephone follow-up for assessment, intervention, and evaluation.  **Materials/Resources**  Not reported | **Standard care**  **Post discharge**  Hospital-based physical training programme within the first 3 weeks after hospital discharge | **4 weeks**  **Predischarge**  1 family meeting  **Post discharge**  Home visits and telephone follow-ups once a week | Baseline,  1 month,  2 months | ADLs (MBI)  QoL (SF-36, WHO-QOL-SRPB)  Depression (CES-D)  Patient satisfaction (PSQ-HK)  Healthcare utilization (hospital readmission and unscheduled Emergency Department attendance rate) |
| Wong 2022, China, RCT | **N = 116 stroke survivors**  IG = 58, mean age 66.21 (SD 10.07) yrs  CG = 58, mean age 67.00 (SD 8.61) yrs  **Type of Stroke (n, %):**  Not reported  **Recruitment setting:**  Neurology units | Structured 4C, home-based post-discharge program  (4C-HBP)  **Provider**  Nurse case manager, physiotherapist, occupational therapist, speech therapist, neurologist and rehabilitation physician | **Predischarge**  Patient and family meetings  - Routine post-discharge advice (blood presssure, medication adherence, dietary, ADL care advice, and rehabilitation exercise).  - Goal setting and preparing for home-based follow-up.  **Post discharge**  Home visits and telephone follow-up for functional training by the NCM under four main themes: (1) teaching, guidance and counseling, (2) treatment and procedures, (3) case management, (4) surveillance. Post discharge program based on the Omaha System.  **Materials/Resources**  - Stroke self-management home care toolkit (e.g. towel, playdough)  - Rehabilitation equipment. (bands, clothespins etc)  - Information booklet. | **Standard care**  **Predischarge**  Advice on blood pressure monitoring,  medication adherence, dietary, daily living care, and rehabilitation exercise  **Post discharge**  Telephone follow up for reinforce the self-care and rehabilitation  exercises. | **12 weeks**  **Predischarge**  One time meeting two days prior to discharge  **Post discharge**  12 weeks: 90 min training per day, 5 days per week | Baseline,  3 months,  6 months | ADLs (MBI)  QoL (EQ-5D-5L)  Stroke Impact scale (SIS)  Self-Efficacy (SEMCD-6) |
| Wong 2024, China, RCT | **N = 116 stroke survivors**  IG = 58, mean age 66.21 (SD 10.07) yrs  CG = 58, mean age 67.00 (SD 8.61) yrs  **Type of Stroke (n, %):**  Not reported  **Recruitment setting:**  Neurology units | Enhanced post discharge home-based care program intervention (EHP)  **Provider**  Nurse (as case manager) supported by an interdisciplinary consultant team | **Predischarge**  Enhanced discharge planning  - physical examination, functional assessment and the Omaha System assessment  - goal setting/care plan  - information sharing and skill training  - current level of stroke-related knowledge, behavior and status evaluation  **Post discharge**  Home visits and telephone follow-ups for comprehensive assessment; treatment and planning; teaching, guidance and counseling; case management; surveillance  **Materials/Resources**  Stroke self-management home care toolkit (written and pictorial instructions for exercise, training, and stroke self-management, daily practice record) and equipment (e.g., arm skate, clothespins, ball, bean bag, and bands). | **Standard care**  **Predischarge**  Discharge care: instructions on medication  regimen, blood pressure and blood sugar monitoring, diet daily  living care, rehabilitation training, drugs  **Post discharge**  Regular medical follow-up. | **12 weeks**  **Predischarge**  1 inpatient visit and 1 coaching session within 48 hours before discharge  **Post discharge**  4 home visits, 4 reinforcement calls, 4 telephone follow-ups, and 24-hour hotline | 3 months,  6 months,  12months | Readmission episode,  Unplanned emergency service |
| Zhou 2019, China, RCT | **N = 244 stroke survivors**  IG = 116, mean age 64.3 (SD 9.2) yrs  CG = 128, mean age 66.2 (SD 9.4) yrs  **N = 244 caregivers**  IG = 116, mean age 51.4 (SD 12.3) yrs  CG = 128, mean age 52.3 (SD 12.8) yrs  **Type of Stroke (n, %):**  IG  Ischemic 85 (73.3%)  CG  Ischemic 104 (81.3%)  **Recruitment setting:**  Neurology units | Rehabilitation Through Caregiver-  Delivered Nurse-Organized Service Programs  **Provider**  Nurse | **Predischarge**  - Task-oriented operation rehabilitation training for patients and caregivers focused on 3 recovery goals: (1) mobility  (transfer, mobility, and stairs); (2) self-care (grooming, feeding, dressing, and bathing);  and (3) continence/ toileting (bowel, bladder, and toilet use).  - Provide information on the usual patterns of poststroke recovery, importance of CV risk factor control, awareness  and implications of low mood, importance of task practice for patients and caregivers  **Post discharge**  Telephone follow-ups to check on patients’ status and problem-solving recommendations  **Materials/Resources**  - Rehabilitation manual (mobility, self-care, continence/toileting)  - Videodisk of the training process | **Standard care**  **Predischarge**  Symptom-targeted medication treatment and regular nursing care without rehabilitation services | **8 weeks**  **Predischarge**  several 15- to 30-minute training sessions  **Post discharge**  3 Telephone calls at week 2, 4, and  8 after discharge | Predischarge,  3 months,  6 months | ADLs (BI)  QoL (EQ-5D)  Caregiver burden (Caregiver Burden Index) |

**Table 4.** Summary Risk of Bias of Included Studies

| Study | Bias domain and signalling question | Description | Risk-of-bias judgement |
| --- | --- | --- | --- |
| **Amiri 2022** | Domain 1: Risk of bias arising from the randomization process | Subjects were randomized by selecting the card and by the patients. Concealed randomization achieved using sealed opaque envelopes. No baseline differences between intervention groups. | Low |
|  | Domain 2: Risk of bias due to deviations from the intended interventions (effect of assignment to intervention) | No blinding of participants and health care providers delivering the interventions. Probably no deviations from the intended intervention. Intention-to-treat (ITT) analyses was used to estimate the effect of assignment to intervention. | Low |
|  | Domain 3: Risk of bias due to missing outcome data | IG=36, CG=36, Not report dropout rate and statistical methods for handling missing outcome data. | Some concern |
|  | Domain 4: Risk of bias in measurement of the outcome | The outcome measurements demonstrated to have good validity, the same measurement methods, and time points. No blinding data collectors. | Some concern |
|  | Domain 5: Risk of bias in selection of the reported result | Published reports include all expected outcomes following a pre-specified plan in outcome measures and analyses listed in the methods section. | Low |
|  |  | **Overall risk of bias for this paper** | **Some concern** |
| **Chen 2018** | Domain 1: Risk of bias arising from the randomization process | Subjects were randomized using a random number table and stored in numbered, sealed, non-transparent envelopes. No baseline differences between intervention groups. | Low |
|  | Domain 2: Risk of bias due to deviations from the intended interventions (effect of assignment to intervention) | No blinding of participants and health care providers delivering the interventions. Probably no deviations from the intended intervention. Intention-to-treat (ITT) analyses was used to estimate the effect of assignment to intervention. | Low |
|  | Domain 3: Risk of bias due to missing outcome data | IG =72 (64 completed, 7 refuse to participate, 1 move to other places), CG=72 (62 completed, 10 refuse to participate), using Generalized Estimating Equation (GEE) model for handling missing outcome data. | Low |
|  | Domain 4: Risk of bias in measurement of the outcome | The outcome measurements demonstrated to have good validity, the same measurement methods, and time points. Well-trained data collectors were blinded to group allocation. | Low |
|  | Domain 5: Risk of bias in selection of the reported result | Published reports include all expected outcomes following a pre-specified plan in outcome measures and analyses listed in the methods section. | Low |
|  |  | **Overall risk of bias for this paper** | **Low** |
| **Chu 2020** | Domain 1: Risk of bias arising from the randomization process | Subjects were randomized using a secure, central, password protected, and internet-based computerized system and using app to inform the participant’s allocation status. No baseline differences between intervention groups. | Low |
|  | Domain 2: Risk of bias due to deviations from the intended interventions (effect of assignment to intervention) | No blinding of participants and health care providers delivering the interventions. Probably no deviations from the intended intervention. Intention-to-treat (ITT) analyses was used to estimate the effect of assignment to intervention. | Low |
|  | Domain 3: Risk of bias due to missing outcome data | IG=31 (26 completed, 1 died, 4 lost follow-up), CG=30 (30 completed), using mixed models for handling missing outcome data. | Low |
|  | Domain 4: Risk of bias in measurement of the outcome | The outcome measurements demonstrated to have good validity, the same measurement methods, and time points. Data collectors were blinded to group allocation. | Low |
|  | Domain 5: Risk of bias in selection of the reported result | Published reports include all expected outcomes following a pre-specified plan in the published study protocol. | Low |
|  |  | **Overall risk of bias for this paper** | **Low** |
| **Farahani 2021** | Domain 1: Risk of bias arising from the randomization process | Subjects were randomized using a random block design. No information about the allocation sequence was concealed. No baseline differences between intervention groups. | Some concern |
|  | Domain 2: Risk of bias due to deviations from the intended interventions (effect of assignment to intervention) | No blinding of participants and health care providers delivering the interventions. Probably no deviations from the intended intervention. A modified intention-to-treat (mITT) analyses was used to estimate the effect of assignment to intervention. | Low |
|  | Domain 3: Risk of bias due to missing outcome data | IG=60 (58 completed, 1 died, 1 stroke), CG=60 (57 completed, 2 died, 1 stroke), using linear regression for handling missing outcome data. | Low |
|  | Domain 4: Risk of bias in measurement of the outcome | The outcome measurements demonstrated to have good validity, the same measurement methods, and time points. Data collectors were probably blinded to group allocation. | Low |
|  | Domain 5: Risk of bias in selection of the reported result | Published reports include all expected outcomes following a pre-specified plan in outcome measures and analyses listed in the methods section. | Low |
|  |  | **Overall risk of bias for this paper** | **Some concern** |
| **Forster 2013** | Domain 1: Risk of bias arising from the randomization process | Cluster randomisation was done centrally at the Clinical Trials Research Unit (CTRU) and used block randomisation. Participants were recruited after randomization and masked to the stroke unit allocation. The groups were well balanced at baseline. | Low |
|  | Domain 2: Risk of bias due to deviations from the intended interventions (effect of assignment to intervention) | Participants were masked to the stroke unit allocation but no blinding staffs delivering the interventions. Probably no deviations from the intended intervention. An intention-to-treat (ITT) analyses was used to estimate the effect of assignment to intervention. | Low |
|  | Domain 3: Risk of bias due to missing outcome data | There were participants recruiting from all clusters. A sensitivity analysis excluding proxy responses was used for handling missing outcome data. | Low |
|  | Domain 4: Risk of bias in measurement of the outcome | The outcome measurements demonstrated to have good validity, the same measurement methods, and time points. Blinding outcome assessors. | Low |
|  | Domain 5: Risk of bias in selection of the reported result | Published reports include all expected outcomes following a pre-specified plan in the published study protocol. | Low |
|  |  | **Overall risk of bias for this paper** | **Low** |
| **Geng 2019** | Domain 1: Risk of bias arising from the randomization process | Subjects were allocated into either control or intervention group depending on which wards they are admitted to. The control and intervention wards were determined by the toss of a coin. No information about the allocation sequence was concealed. No baseline differences between intervention groups. | High |
|  | Domain 2: Risk of bias due to deviations from the intended interventions (effect of assignment to intervention) | No blinding of participants and health care providers delivering the interventions. Probably no deviations from the intended intervention. Intention-to-treat (ITT) analyses was used to estimate the effect of assignment to intervention. | Low |
|  | Domain 3: Risk of bias due to missing outcome data | IG=30 (29 completed, 1 admission due to kidney failure), CG=30 (28 completed, 1 admission due to heart failure, 1 could not be contacted). A linear mixed model was used for handling missing outcome data. | Low |
|  | Domain 4: Risk of bias in measurement of the outcome | The outcome measurements demonstrated to have good validity, the same measurement methods, and time points. Well-trained data collectors were blinded to group allocation. | Low |
|  | Domain 5: Risk of bias in selection of the reported result | Published reports include all expected outcomes following a pre-specified plan in outcome measures and analyses listed in the methods section. | Low |
|  |  | **Overall risk of bias for this paper** | **High** |
| **Hui 2012** | Domain 1: Risk of bias arising from the randomization process | Subjects were randomized. No information about the allocation sequence was concealed. No baseline differences between intervention groups. | Some concern |
|  | Domain 2: Risk of bias due to deviations from the intended interventions (effect of assignment to intervention) | No blinding of participants and health care providers delivering the interventions. Probably no deviations from the intended intervention. A modified intention-to-treat (mITT) analyses was used to estimate the effect of assignment to intervention. | Low |
|  | Domain 3: Risk of bias due to missing outcome data | IG=30 (27 completed, 1 lost follow-up, 2 recover), CG=30 (26 completed, 2 lost follow-up, 2 refuse). No report about statistical methods for handling missing outcome data. | Some concern |
|  | Domain 4: Risk of bias in measurement of the outcome | The outcome measurements demonstrated to have good validity, the same measurement methods, and time points. No blinding data collectors. | Some concern |
|  | Domain 5: Risk of bias in selection of the reported result | Published reports include all expected outcomes following a pre-specified plan in outcome measures and analyses listed in the methods section. | Low |
|  |  | **Overall risk of bias for this paper** | **Some concern** |
| **Jafari 2020** | Domain 1: Risk of bias arising from the randomization process | Subjects were randomized using color card methodology. No information about the allocation sequence was concealed. No baseline differences between intervention groups. | Some concern |
|  | Domain 2: Risk of bias due to deviations from the intended interventions (effect of assignment to intervention) | No blinding of participants and health care providers delivering the interventions. Probably no deviations from the intended intervention. Intention-to-treat (ITT) analyses was used to estimate the effect of assignment to intervention. | Low |
|  | Domain 3: Risk of bias due to missing outcome data | IG=30, CG=30, No report about missing outcome data and the statistical methods for handling missing outcome data. | Some concern |
|  | Domain 4: Risk of bias in measurement of the outcome | The outcome measurements demonstrated to have good validity, the same measurement methods, and time points. No blinding data collectors. | Some concern |
|  | Domain 5: Risk of bias in selection of the reported result | Published reports include all expected outcomes following a pre-specified plan in outcome measures and analyses listed in the methods section. | Low |
|  |  | **Overall risk of bias for this paper** | **Some concern** |
| **Lin 2022** | Domain 1: Risk of bias arising from the randomization process | Subjects were randomized using the IBM SPSS Statistics version 25 random sampling, concealed in sequentially opaque and sealed envelopes. No baseline differences between intervention groups. | Low |
|  | Domain 2: Risk of bias due to deviations from the intended interventions (effect of assignment to intervention) | No blinding of participants and health care providers delivering the interventions. Probably no deviations from the intended intervention. Intention-to-treat (ITT) analyses was used to estimate the effect of assignment to intervention. | Low |
|  | Domain 3: Risk of bias due to missing outcome data | IG=70 (69 completed, 1 died), CG=70 (65 completed, 2 lost, 3 died), using linear mixed effect models for handling missing outcome data. | Low |
|  | Domain 4: Risk of bias in measurement of the outcome | The outcome measurements demonstrated to have good validity, the same measurement methods, and time points, except the number of adverse events and unplanned rehospitalisation. Data collectors were blinded to treatment allocation. | High |
|  | Domain 5: Risk of bias in selection of the reported result | Published reports include all expected outcomes following a pre-specified plan in the published study protocol. | Low |
|  |  | **Overall risk of bias for this paper** | **High** |
| **Liu 2018** | Domain 1: Risk of bias arising from the randomization process | Subjects were randomized. No information about the allocation sequence was concealed. No baseline differences between intervention groups. | Some concern |
|  | Domain 2: Risk of bias due to deviations from the intended interventions (effect of assignment to intervention) | No blinding of participants and health care providers delivering the interventions. Probably no deviations from the intended intervention. Intention-to-treat (ITT) analyses was used to estimate the effect of assignment to intervention. | Low |
|  | Domain 3: Risk of bias due to missing outcome data | IG=20, CG=20, No report about missing outcome data and statistical methods for handling missing outcome data. | Some concern |
|  | Domain 4: Risk of bias in measurement of the outcome | The outcome measurements demonstrated to have good validity, the same measurement methods, and time points. No blinding data collectors. | Some concern |
|  | Domain 5: Risk of bias in selection of the reported result | Published reports include all expected outcomes following a pre-specified plan in outcome measures and analyses listed in the methods section. | Low |
|  |  | **Overall risk of bias for this paper** | **Some concern** |
| **Qian 2019** | Domain 1: Risk of bias arising from the randomization process | Subjects were randomized. No information about the allocation sequence was concealed. No baseline differences between intervention groups. | Some concern |
|  | Domain 2: Risk of bias due to deviations from the intended interventions (effect of assignment to intervention) | No blinding of participants and health care providers delivering the interventions. Probably no deviations from the intended intervention. Intention-to-treat (ITT) analyses was used to estimate the effect of assignment to intervention. | Low |
|  | Domain 3: Risk of bias due to missing outcome data | IG=35, CG=37, Missing data were substituted by the average or mode value, no report about statistical methods for handling missing outcome data. | Some concern |
|  | Domain 4: Risk of bias in measurement of the outcome | The outcome measurements demonstrated to have good validity, the same measurement methods, and time points, except utilization of health services. No information about blinding data collectors. | High |
|  | Domain 5: Risk of bias in selection of the reported result | Published reports include all expected outcomes following a pre-specified plan in outcome measures and analyses listed in the methods section. | Low |
|  |  | **Overall risk of bias for this paper** | **High** |
| **Shyu 2010** | Domain 1: Risk of bias arising from the randomization process | Subjects were randomized. No information about the allocation sequence was concealed. No baseline differences between intervention groups. | Some concern |
|  | Domain 2: Risk of bias due to deviations from the intended interventions (effect of assignment to intervention) | No blinding of participants and health care providers delivering the interventions. Probably no deviations from the intended intervention. Intention-to-treat (ITT) analyses was used to estimate the effect of assignment to intervention. | Low |
|  | Domain 3: Risk of bias due to missing outcome data | IG=72 (61 completed, 6 refuse to participate, 5 died), CG=86 (75 completed, 6 refuse to participate, 5 died), using Generalized Estimating Equation (GEE) model for handling missing outcome data. | Low |
|  | Domain 4: Risk of bias in measurement of the outcome | The outcome measurements demonstrated to have good validity, the same measurement methods, and time points, except utilization of health services. No information about blinding data collectors. | High |
|  | Domain 5: Risk of bias in selection of the reported result | Published reports include all expected outcomes following a pre-specified plan in outcome measures and analyses listed in the methods section. | Low |
|  |  | **Overall risk of bias for this paper** | **High** |
| **Torres-Arreola 2009** | Domain 1: Risk of bias arising from the randomization process | Subjects were randomized using consecutive opaque envelopes. No baseline differences between intervention groups. | Low |
|  | Domain 2: Risk of bias due to deviations from the intended interventions (effect of assignment to intervention) | No blinding of participants and health care providers delivering the interventions. Probably no deviations from the intended intervention. Intention-to-treat (ITT) analyses was used to estimate the effect of assignment to intervention. | Low |
|  | Domain 3: Risk of bias due to missing outcome data | IG=59 (32 completed, 4 died, 23 drop out), CG=51 (35 completed, 1 died, 15 drop out)  IG=36, CG=36, No report about statistical methods for handling missing outcome data. | Some concern |
|  | Domain 4: Risk of bias in measurement of the outcome | The outcome measurements demonstrated to have good validity, the same measurement methods, and time points. Data collectors were blinded to group allocation. | Low |
|  | Domain 5: Risk of bias in selection of the reported result | Published reports include all expected outcomes following a pre-specified plan in outcome measures and analyses listed in the methods section. | Low |
|  |  | **Overall risk of bias for this paper** | **Some concern** |
| **Wong 2015** | Domain 1: Risk of bias arising from the randomization process | Subjects were randomized using a computer-generated randomized block design, concealed in sequential opaque and sealed envelopes. No baseline differences between intervention groups. | Low |
|  | Domain 2: Risk of bias due to deviations from the intended interventions (effect of assignment to intervention) | No blinding of participants and health care providers delivering the interventions. Probably no deviations from the intended intervention. Intention-to-treat (ITT) analyses was used to estimate the effect of assignment to intervention. | Low |
|  | Domain 3: Risk of bias due to missing outcome data | IG=54 (50 completed, 1 move to another cluster, 1 refuse, 1 cognitive detoriation, 1 contaminate by other therapy), CG=54 (50 completed, 3 move to China, 2 refuse), missing data for ordinal education and continuous age were replaced by subgroup medians and means respectively. | Low |
|  | Domain 4: Risk of bias in measurement of the outcome | The outcome measurements demonstrated to have good validity, the same measurement methods, and time points. Trained data collectors were blinded to group allocation. | Low |
|  | Domain 5: Risk of bias in selection of the reported result | Published reports include all expected outcomes following a pre-specified plan in outcome measures and analyses listed in the methods section. | Low |
|  |  | **Overall risk of bias for this paper** | **Low** |
| **Wong 2022** | Domain 1: Risk of bias arising from the randomization process | Subjects were randomized using a computer-generated randomized block design, concealed in sequential opaque and sealed envelopes. No baseline differences between intervention groups. | Low |
|  | Domain 2: Risk of bias due to deviations from the intended interventions (effect of assignment to intervention) | No blinding of participants and health care providers delivering the interventions. Probably no deviations from the intended intervention. Intention-to-treat (ITT) analyses was used to estimate the effect of assignment to intervention. | Low |
|  | Domain 3: Risk of bias due to missing outcome data | IG=58 (56 completed, 2 died), CG=58 (55 completed, 2 refuse to participation, 1 could not be contacted), using Generalized Estimating Equation (GEE) model for handling missing outcome data. | Low |
|  | Domain 4: Risk of bias in measurement of the outcome | The outcome measurements demonstrated to have good validity, the same measurement methods, and time points. Data collectors were blinded to group allocation. | Low |
|  | Domain 5: Risk of bias in selection of the reported result | Published reports include all expected outcomes following a pre-specified plan in outcome measures and analyses listed in the methods section. | Low |
|  |  | **Overall risk of bias for this paper** | **Low** |
| **Wong 2024** | Domain 1: Risk of bias arising from the randomization process | Subjects were randomized using a computer-generated randomized block design, concealed in sequential opaque and sealed envelopes. No baseline differences between intervention groups. | Low |
|  | Domain 2: Risk of bias due to deviations from the intended interventions (effect of assignment to intervention) | No blinding of participants and health care providers delivering the interventions. Probably no deviations from the intended intervention. Intention-to-treat (ITT) analyses was used to estimate the effect of assignment to intervention. | Low |
|  | Domain 3: Risk of bias due to missing outcome data | IG=58, CG=58, No report about missing outcome data and statistical methods for handling missing outcome data. | Some concern |
|  | Domain 4: Risk of bias in measurement of the outcome | The outcome measurements demonstrated to have good validity, the same measurement methods, and time points, except utilization of health services. Well-trained data collectors were blinded to group allocation. | High |
|  | Domain 5: Risk of bias in selection of the reported result | Published reports include all expected outcomes following a pre-specified plan in outcome measures and analyses listed in the methods section. | Low |
|  |  | **Overall risk of bias for this paper** | **High** |
| **Zhou 2019** | Domain 1: Risk of bias arising from the randomization process | Subjects were randomized using a random block, secure, central, password protected, and internet-based computerized system and using app to inform the participant’s allocation status. No baseline differences between intervention groups. | Low |
|  | Domain 2: Risk of bias due to deviations from the intended interventions (effect of assignment to intervention) | No blinding of participants and health care providers delivering the interventions. Probably no deviations from the intended intervention. Intention-to-treat (ITT) analyses was used to estimate the effect of assignment to intervention. | Low |
|  | Domain 3: Risk of bias due to missing outcome data | IG=116 (108 completed, 8 missing), CG=128 (125 completed, missing 3), using mixed models for handling missing outcome data. | Low |
|  | Domain 4: Risk of bias in measurement of the outcome | The outcome measurements demonstrated to have good validity, the same measurement methods, and time points. Trained data collectors were blinded to group allocation. | Low |
|  | Domain 5: Risk of bias in selection of the reported result | Published reports include all expected outcomes following a pre-specified plan in the published study protocol. | Low |
|  |  | **Overall risk of bias for this paper** | **Low** |

**Table 5.** Risk of Bias of Included Studies for each outcome

 Low risk

 Some concerns

 High risk

A Risk of bias arising from the randomization process

B Risk of bias due to deviations from the intended interventions

C Risk of bias due to missing outcome data

D Risk of bias in measurement of the outcome

E Risk of bias in selection of the reported result

| Study | Outcome | A | B | C | D | E | Overall |
| --- | --- | --- | --- | --- | --- | --- | --- |
| Amiri 2022 | Self-Efficacy_SSEQ |  |  |  |  |  |  |
| Chen 2018 | ADL_BI |  |  |  |  |  |  |
| Chen 2018 | Quality of life_SF-36 |  |  |  |  |  |  |
| Chen 2018 | Depression_CES-D-C |  |  |  |  |  |  |
| Chen 2018 | Self-Efficacy_SSEQ |  |  |  |  |  |  |
| Chen 2018 | Readmission |  |  |  |  |  |  |
| Chu 2020 | ADL_BI |  |  |  |  |  |  |
| Chu 2020 | Quality of life_ EQ-5D |  |  |  |  |  |  |
| Chu 2020 | Caregiver Burden_CBI |  |  |  |  |  |  |
| Farahani 2021 | Caregiver Burden_CBI |  |  |  |  |  |  |
| Forster 2013 | ADL_BI |  |  |  |  |  |  |
| Forster 2013 | ADL_NEADL |  |  |  |  |  |  |
| Forster 2013 | Quality of life_ EQ-5D |  |  |  |  |  |  |
| Forster 2013 | Quality of life_ SIS |  |  |  |  |  |  |
| Forster 2013 | Depression_HADS-D |  |  |  |  |  |  |
| Forster 2013 | Caregiver’s Quality of life_EQ-5D |  |  |  |  |  |  |
| Geng 2019 | ADL_MBI |  |  |  |  |  |  |
| Geng 2019 | Quality of life_SF-36 |  |  |  |  |  |  |
| Geng 2019 | Stroke-relate health knowledge_ Stroke Knowledge Questionnaire |  |  |  |  |  |  |
| Hui 2012 | ADL_MBI |  |  |  |  |  |  |
| Jafari 2020 | Quality of life_ SS-QOL |  |  |  |  |  |  |
| Wong 2022 | ADL_MBI |  |  |  |  |  |  |
| Wong 2022 | Quality of life_SIS |  |  |  |  |  |  |
| Wong 2022 | Quality of life_EQ-5D-5L |  |  |  |  |  |  |
| Wong 2022 | Self-Efficacy_SEMCD-6 |  |  |  |  |  |  |
| Lin 2022 | Quality of life_ SSQoL-12 |  |  |  |  |  |  |
| Lin 2022 | Self-Efficacy_SSEQ |  |  |  |  |  |  |
| Lin 2022 | Stroke-relate health knowledge_ SPKQ |  |  |  |  |  |  |
| Lin 2022 | Caregiver Burden_CSI |  |  |  |  |  |  |
| Lin 2022 | Readmission |  |  |  |  |  |  |
| Liu 2018 | Quality of life_SF-36 |  |  |  |  |  |  |
| Qian 2019 | ADL_BI |  |  |  |  |  |  |
| Qian 2019 | Patient’s Satisfaction with care_ PSHC |  |  |  |  |  |  |
| Qian 2019 | Readmission |  |  |  |  |  |  |
| Qian 2019 | Emergency visit |  |  |  |  |  |  |
| Shyu 2010 | ADL_BI |  |  |  |  |  |  |
| Shyu 2010 | Quality of life_SF-36 |  |  |  |  |  |  |
| Shyu 2010 | Caregiver’s Quality of life_SF-36 |  |  |  |  |  |  |
| Shyu 2010 | Readmission |  |  |  |  |  |  |
| Torres-Arreola 2009 | ADL_BI |  |  |  |  |  |  |
| Wong 2024 | Readmission |  |  |  |  |  |  |
| Wong 2024 | Emergency visit |  |  |  |  |  |  |
| Wong 2015 | ADL_MBI |  |  |  |  |  |  |
| Wong 2015 | Quality of life_SF-36 |  |  |  |  |  |  |
| Wong 2015 | Depression_ CES-D |  |  |  |  |  |  |
| Wong 2015 | Patient’s Satisfaction with care_ PSQ-HK |  |  |  |  |  |  |
| Wong 2015 | Readmission |  |  |  |  |  |  |
| Wong 2015 | Emergency visit |  |  |  |  |  |  |
| Zhou 2019 | ADL_BI |  |  |  |  |  |  |
| Zhou 2019 | Quality of life_EQ-5D |  |  |  |  |  |  |
| Zhou 2019 | Caregiver Burden_CBI |  |  |  |  |  |  |

**Table 6.** Summary of intervention components and underpinning theories

| Components in Transitional care | | | | | | Theory |
| --- | --- | --- | --- | --- | --- | --- |
| Study | Patient- and caregiver-centred assessment | Goal setting and personalized care planning | Health education | Functional rehabilitation | Self-management |  |
| Amiri 2022 | *** | ** | *** | X | *** | 5A self-management model |
| Chen 2018 | *** | * | *** | * | *** | Health Empowerment Model |
| Chu 2020 | X | * | X | *** | X | X |
| Farahani 2021 | X | X | *** | X | X | X |
| Forster 2013 | *** | X | *** | X | X | X |
| Geng 2019 | ** | X | ** | X | X | Integrated Behavioural Model |
| Hui 2012 | X | ** | *** | *** | X | X |
| Jafari 2020 | X | X | *** | X | X | X |
| Lin 2022 | X | ** | *** | * | X | Self-Efficacy Theory |
| Liu 2018 | *** | ** | * | ** | X | Naylor Transitional Care Model |
| Qian 2019 | X | X | *** | ** | X | Omaha System, Brunnstrom’s stages of recovery |
| Shyu 2010 | * | X | * | X | X | X |
| Torres-Arreola 2009 | X | X | *** | *** | X | Brunnstrom’s stages of recovery |
| Wong 2015 | *** | *** | X | X | *** | Omaha System, Naylor Transitional Care Model |
| Wong 2022 | *** | ** | *** | * | * | Omaha System, Transitional Care Framework |
| Wong 2024 | *** | ** | * | X | * | Omaha System |
| Zhou 2019 | X | *** | *** | *** | X | Motor Relearning Theory |

Key: * = if only the component’s name or little detailed explanation was reported; **= if the component’s name and some explanation about the component were reported; *** = if the component’s name, some detailed explanation and example of content provided were reported; X = if not reported in the intervention description.

**Supplementary Figure**

**Forest Plot of activity of daily living: Transitional care intervention vs. Control**

Figure 1. Activity of daily living at 6 months

**Forest Plot of quality of life: Transitional care intervention vs. Control**

Figure 2. Mental component score (MCS) at 3 months

Figure 3. Physical component score (PCS) at 1 month

Figure 4. Physical component score (PCS) at 3 months

Figure 5. Physical function component score at 6 months

Figure 6. Bodily pain component score at 6 months

Figure 7. Vitality component score at 6 months

Figure 8. Social functioning component score at 6 months

Figure 9. Role limitations due to emotional problems component score at 6 months

Figure 10. Mental health component score at 6 months

**Forest Plot of depression: Transitional care intervention vs. Control**

Figure 11. Depression at 1 month

**Forest Plot of self-efficacy: Transitional care intervention vs. Control**

Figure 12. Self-Efficacy at 3 months

Figure 13. Self-Efficacy at 6 months

**Forest Plot of** **stroke-related knowledge: Transitional care intervention vs. Control**

Figure 14. Stroke-related knowledge at 3 months

Figure 15. Stroke-related knowledge at 6 months

**Forest Plot of** **patient satisfaction with care: Transitional care intervention vs. Control**

Figure 16. Patient satisfaction with care up to 3 months

**Forest Plot of** **caregiver burden: Transitional care intervention vs. Control**

Figure 17. Caregiver burden at 6 months

**Forest Plot of** **healthcare utilisation: Transitional care intervention vs. Control**

Figure 18. Hospital readmission at 6 months

Figure 19. Emergency visits up to 3 months

**Sensitivity analysis**

1. **Activity of daily living**

Low risk

Some concerns

High risk

A Risk of bias arising from the randomization process

B Risk of bias due to deviations from the intended interventions

C Risk of bias due to missing outcome data

D Risk of bias in measurement of the outcome

E Risk of bias in selection of the reported result

|  | A | B | C | D | E | Overall |
| --- | --- | --- | --- | --- | --- | --- |
| Chen 2018 |  |  |  |  |  |  |
| Hui 2012 |  |  |  |  |  |  |
| Shyu 2010 |  |  |  |  |  |  |
| Wong 2015 |  |  |  |  |  |  |

Figure S1. Risk of Bias for Outcome: Activity of daily living at 1 month

Figure S2. Sensitivity analysis Risk of Bias for Outcome: Activity of daily living at 1 month

|  | A | B | C | D | E | Overall |
| --- | --- | --- | --- | --- | --- | --- |
| Chen 2018 |  |  |  |  |  |  |
| Chu 2020 |  |  |  |  |  |  |
| Hui 2012 |  |  |  |  |  |  |
| Qian 2019 |  |  |  |  |  |  |
| Shyu 2010 |  |  |  |  |  |  |
| Wong 2022 |  |  |  |  |  |  |
| Zhou 2019 |  |  |  |  |  |  |

Figure S3. Risk of Bias for Outcome: Activity of daily living at 3 months

Figure S4. Sensitivity analysis Risk of Bias for Outcome: Activity of daily living at 3 months

2. **Quality of Life**

All studies had Some concerns or overall High ROB (Figure I) therefore sensitivity analysis not appropriate.

|  | D1 | D2 | D3 | D4 | D5 | Overall |
| --- | --- | --- | --- | --- | --- | --- |
| Geng 2019 |  |  |  |  |  |  |
| Liu 2018 |  |  |  |  |  |  |
| Shyu 2010 |  |  |  |  |  |  |

Figure S5. Risk of Bias for Outcome: Quality of Life at 6 months

3. **Patient satisfaction with care**

|  | A | B | C | D | E | Overall |
| --- | --- | --- | --- | --- | --- | --- |
| Qian 2019 |  |  |  |  |  |  |
| Wong 2015 |  |  |  |  |  |  |

Figure S6. Risk of Bias for Outcome: Patient satisfaction with care

Figure S7. Sensitivity analysis Risk of Bias for Outcome: Patient satisfaction with care up to 3 months

4. **Hospital readmission**

|  | D1 | D2 | D3 | D4 | D5 | Overall |
| --- | --- | --- | --- | --- | --- | --- |
| Chen 2018 |  |  |  |  |  |  |
| Qian 2019 |  |  |  |  |  |  |
| Wong 2015 |  |  |  |  |  |  |

Figure S8. Risk of Bias for Outcome: Hospital readmission up to 3 months

Figure S9. Sensitivity analysis Risk of Bias for Outcome: Hospital readmission up to 3 months

**Subgroup analysis was carried out for Activity of daily living and hospital readmission to identify variances based on**:

1. Duration of intervention
2. Theoretical underpinning
3. Mode of contact after discharge
4. Intervention provider
5. Intensity of contact after discharge
6. Intervention components
1. **Activity of daily living at 3 months**

Figure G1.1 Duration of intervention: No effect

Figure G1.2 Theoretical underpinning: No effect

Figure G1.3 Mode of contact after discharge: Favours a combination of home visit and phone call

Figure G1.4 Intervention provider: No effect

Figure G1.5 Intensity of contact after discharge: No effect

Figure G1.6 Intervention components: Favours studies that had 4-5 intervention components

2. **Hospital readmission up to 3 months**

Figure G2.1 Mode of contact after discharge: No effect

Figure 2.2 Intensity of contact after discharge: No effect

Figure G2.3 Intervention components: No effect

Duration of intervention, Theoretical underpinning, Intervention provider: not appropriate to analyse

**Table 7. GRADE summary of findings**

**Summary of findings: Nurse-initiated Transitional care intervention compared to usual care for older stroke survivors at transitions from hospital to home**

**Patient or population:** older stroke survivors

**Setting:** transitions from hospital to home

**Intervention:** nurse-initiated transitional care intervention

**Comparison:** usual care

| **Certainty assessment** | | | | | | | **№ of patients** | | **Effect** | | **Certainty** | **Importance** |
| --- | --- | --- | --- | --- | --- | --- | --- | --- | --- | --- | --- | --- |
| **№ of studies** | **Study design** | **Risk of bias** | **Inconsistency** | **Indirectness** | **Imprecision** | **Other considerations** | **Transitional care intervention** | **Usual care** | **Relative (95% CI)** | **Absolute (95% CI)** |  |  |
| **ADL at 1 month** | | | | | | | | | | | | |
| 4 | randomised trials | serious^a^ | serious^b^ | not serious | serious^c^ | none | 225 | 238 | - | SMD **0.54 higher** (0.12 higher to 0.97 higher) | ⨁◯◯◯ Very low^a,b,c^ |  |
| **ADL at 3 months** | | | | | | | | | | | | |
| 7 | randomised trials | serious^d^ | serious^e^ | not serious | serious^f^ | none | 411 | 437 | - | SMD **0.43 higher** (0.12 higher to 0.74 higher) | ⨁◯◯◯ Very low^d,e,f^ |  |
| **Mental component score (MCS) at 1 month** | | | | | | | | | | | | |
| 2 | randomised trials | not serious | not serious | not serious | very serious^g^ | none | 126 | 126 | - | MD **0.73 higher** (0.34 higher to 1.12 higher) | ⨁⨁◯◯ Low^g^ |  |
| **Role limitations due to physical problems component score at 6 months** | | | | | | | | | | | | |
| 3 | randomised trials | serious^h^ | not serious | not serious | very serious^i^ | none | 122 | 136 | - | MD **7.7 higher** (0.17 higher to 15.23 higher) | ⨁◯◯◯ Very low^h,i^ |  |
| **General health component score at 6 months** | | | | | | | | | | | | |
| 3 | randomised trials | serious^j^ | not serious | not serious | very serious^k^ | none | 122 | 136 | - | MD **5.32 higher** (0.87 higher to 9.77 higher) | ⨁◯◯◯ Very low^j,k^ |  |
| **Patient satisfaction with care up to 3 months** | | | | | | | | | | | | |
| 2 | randomised trials | not serious | serious^l^ | not serious | very serious^m^ | none | 89 | 91 | - | SMD **3.62 higher** (1.08 higher to 6.17 higher) | ⨁◯◯◯ Very low^l,m^ |  |
| **Hospital readmission up to 3 months** | | | | | | | | | | | | |
| 3 | randomised trials | serious^n^ | not serious | not serious | very serious^o^ | none | 15/161 (9.3%) | 29/163 (17.8%) | **RR 0.54** (0.30 to 0.97) | **82 fewer per 1000** (from 125 fewer to 5 fewer) | ⨁◯◯◯ Very low^n,o^ |  |

**CI:** confidence interval; **MD:** mean difference; **RR:** risk ratio; **SMD:** standardised mean difference

**Explanations**

^a^Downgraded by 1 level due to 1) no information about the concealment of allocation sequence in two studies, 2) blinding was not possible due to the nature of the interventions, 3) incomplete outcome data in two studies with one did not report statistical analyses for handling missing outcome data leading to some concerns, which contributes to 2/4 to the overall body of evidence (47.3% weight in the meta-analysis).

^b^Downgraded by 1 level due to a high I² value (79%); two of the four estimates favoured the intervention, while the other two showed no difference; three of the four studies' estimates lie between 0.1 and 0.7, and only one study is greater than 1.2, engendering a conclusion of minimal inconsistency.

^c^Downgraded by 1 level due to a sample size of less than 2,000, but more than 50% of the optimal information size (i.e., 400 overall).

^d^Downgraded by 1 level due to 1) no information about the concealment of allocation sequence in three studies, 2) blinding was not possible due to the nature of the interventions, 3) incomplete outcome data in three studies with two did not report statistical analyses for handling missing outcome data leading to some concerns, which contributes to 3/7 to the overall body of evidence (39.7% weight in the meta-analysis).

^e^Downgraded by 1 level due to a high I² value (78%); three of the seven estimates favoured treatment, while four showed no difference; six of the seven study estimates lie between 0.1 and 0.5, with only one exceeding 1.5, suggesting minimal inconsistency.

^f^Downgraded by 1 level due to a sample size of less than 2,000, but more than 50% of the optimal information size (400 overall).

^g^Downgraded by 2 levels due to a sample size of less than 2,000, and less than 50% of the optimal information size (400 overall).

^h^Downgraded by 1 level due to 1) high risk of randomisation and concealment of allocation in one study and some concern in two studies, 2) incomplete outcome data in three trials, with one of these did not report statistical analyses for handling missing outcome data, leading to some concern, and 3) no blinding of data collectors in two studies.

^i^Downgraded by 2 levels for imprecision due to a sample size of less than 2,000, and less than 50% of the optimal information size (400 overall).

^j^Downgraded by 1 level due to 1) high risk of randomisation and concealment of allocation in one study and some concern in two studies, 2) incomplete outcome data in three trials, with one of these did not report statistical analyses for handling missing outcome data, leading to some concern, and 3) no blinding of data collectors in two studies.

^k^Downgraded by 2 levels for imprecision due to a sample size of less than 2,000, and less than 50% of the optimal information size (400 overall).

^l^Downgraded by 1 level due to a high percentage of I² (96%).

^m^Downgraded by 2 levels due to a sample size of less than 2,000 and less than 50% of the optimal information size (400 overall).

^n^Downgraded by 1 level due to slight concern about the concealment of allocation sequence, no information about handling missing outcome data, no blinding of data collectors, and different time points measured in the utilisation of health services.

^o^Downgraded by 2 levels due to the ratio of the upper to the lower boundary of the CI being more than 3 (3.24).
